# Consumer perspectives of accessing medicinal cannabis treatment from cannabis clinics versus generalist health settings in Australia

**DOI:** 10.1186/s42238-025-00338-z

**Published:** 2025-10-27

**Authors:** Nicholas Lintzeris, Jonathon C Arnold, Iain S McGregor, Llewellyn Mills

**Affiliations:** 1 Specialty of Addiction Medicine, Faculty Medicine and Health, University of Sydney, c/o 591 South Dowling Street, Surry Hills, NSW 2010 Australia; 2 Drug and Alcohol Services, South Eastern Sydney Local Health District, Sydney, NSW Australia; 3 Drug and Alcohol Clinical Research and Improvement Network (DACRIN), Sydney, NSW Australia; 4 Lambert Initiative for Cannabinoid Therapeutics, The University of Sydney, Sydney, NSW Australia; 5 Faculty of Science, School of Psychology, The University of Sydney, Sydney, NSW Australia; 6 Brain and Mind Centre, The University of Sydney, Sydney, NSW Australia; 7 Centre for Human Psychopharmacology, Swinburne University of Technology, Hawthorn, VIC Australia

**Keywords:** Medicinal cannabis, Medical cannabis, Cannabis clinics, Consumer, Generalist health

## Abstract

**Objectives:**

To examine consumer perspectives regarding medicinal cannabis treatment provided to patients attending generalist health settings (GHS) compared to those attending medicinal cannabis clinics (MCC).

**Methods:**

An anonymous online survey, with convenience sampling of adults self-reporting prescribed medicinal cannabis in the preceding 12 months. Outcomes included participant characteristics, indications and patterns of cannabis use, consumer satisfaction.

**Results:**

Most participants (1899/2394, 79.3%) accessed treatment from MCC. Compared to generalist settings, MCC participants were younger (3.5 years, 95%CI: -4.7, -2.2), less likely to be prescribed oral products (OR = 0.4, 0.4–0.5), and had higher rates of employment (OR = 2.1, 1.8–2.5) and cannabis use disorder (OR = 1.5, 1.2–1.8). MCC participants were less likely to seek treatment for chronic pain (OR = 0.7, 0.6–0.8), but more likely for mental health conditions (OR = 1.6, 1.3-2.0). MCC participants had lower satisfaction levels regarding consultation duration (OR = 0.8, 0.7–0.9), information about potential harms and benefits (OR = 0.7, 0.6–0.9), use of other treatment approaches (OR = 0.5, 0.4–0.6) and treatment costs (OR = 0.6, 0.5–0.7).

**Conclusion:**

We identified differences in the profile of patients, type of treatment provided between service settings, and there were higher levels of satisfaction amongst consumers accessing treatment in GHS settings. Further research is required to examine other dimensions of quality and appropriateness of care provided in MCC.

**Supplementary Information:**

The online version contains supplementary material available at 10.1186/s42238-025-00338-z.

## Introduction

Medicinal cannabis products have been legally available in Australia since 2016, prescribed by a medical (or nurse) practitioner and dispensed at a pharmacy [1]. Most medicinal cannabis products are unapproved by the Therapeutics Goods Administration, the government department responsible for regulating medicines in Australia, and are prescribed under Special Access or Authorised Prescriber Schemes allowing compassionate use of unregistered medicines in Australia [1, 2]. Medical or nurse prescribers must obtain Commonwealth government approval through these schemes in order to prescribe medicinal cannabis products [3].

There were relatively few patient approvals made by the TGA in the first few years after legalisation [2, 4]. A Parliamentary Senate Inquiry in 2020 concluded that the reluctance of medical practitioners to prescribe medicinal cannabis was a significant barrier to patient access [5]. Surveys of people using medical cannabis in Australia in 2018 [6] and 2020 [7] indicated that only 2% (25/1044) and 36% (601/1600) of respondents had accessed prescribed medicinal cannabis respectively, with the remainder using illicit cannabis products. Note that in this paper, medical cannabis refers to prescribed and illicit sources of cannabis used for medical reasons, whereas medicinal cannabis is restricted to legally prescribed cannabis products. When queried, 48% (433/906) of participants in 2018 [6] and 36% (358/997) in 2020 [7] indicated they had not accessed prescribed medicinal cannabis because they did not know a medical practitioner willing to prescribe.

A number of barriers to health practitioner engagement in medicinal cannabis treatment have been documented [8–10]. Many practitioners are uncomfortable or unwilling to prescribe medicinal cannabis due to poor knowledge of clinical and pharmacological features of medicinal cannabis products, limited training in medicinal cannabis, insufficient evidence to support use for many health conditions, concerns regarding adverse effects and patient harm, burdensome regulatory requirements, medico-legal concerns, and stigma within the medical profession against patients and practitioners involved with medicinal cannabis treatment.

The unwillingness of many practitioners to prescribe medicinal cannabis, alongside strong consumer demand created the conditions for the establishment and expansion of medicinal cannabis clinics (MCC) in Australia and other countries [11]. MCC provide medicinal cannabis treatment for a diverse range health conditions to patients outside of their normal or generalist health setting (GHS). In Australia, the emergence of such clinics also coincided with the normalisation of telehealth medicine in the aftermath of the COVID-19 pandemic [12], such that patients from anywhere in Australia could access medicinal cannabis treatment by telehealth, with medicines either delivered in the mail or dispensed at local pharmacies. In some circumstances, there appears to be vertical integration - with medicinal cannabis producers operating MCC that employ doctors, nurses and pharmacists in these clinics, and encourage prescription of products supplied by the producers [13].

A number of concerns have been identified with the emergence of MCC [10, 11, 13]. These include: the fragmentation of health care, over-reliance on a single treatment option for complex health conditions, high patient fees, and the potential for conflicts of interest for healthcare practitioners employed by medicinal cannabis companies [10, 11, 13]. Some medical practitioners have described negative attitudes towards medicinal cannabis prescribers, particularly doctors working in MCC, as summarised by Dobson and colleagues [10]: “many in the medical community did not consider medicinal cannabis as a legitimate therapeutic option and viewed prescribers as engaging in pseudo-recreational or unsound medical practices” (p 1286). In contrast, supporters of MCC highlight that they provide a service to consumers: responding to patient demand in light of the reluctance of most medical practitioners to prescribe; they provide a non-judgemental service to consumers who may have encountered prejudices against medicinal cannabis; and that working in such ‘high volume’ medicinal cannabis treatment settings enhances the expertise of practitioners in providing medicinal cannabis treatment [10, 13].

The emergence and expansion of MCC in Australia has most likely facilitated the marked increase in patients accessing medicinal cannabis, with over a hundred thousand SAS-B applications for medicinal cannabis products each calendar year since 2020 [2]. However, to date there has been little research in Australia examining patient perspectives of accessing treatment from MCC [10]. Australian studies of consumer perspectives have identified barriers to patients accessing medicinal cannabis arising from difficulties in finding medical practitioners willing to prescribe, the cost of medications, stigma (from some healthcare providers, friends and family) and restrictions to driving of motor vehicles [6, 7, 14, 15]. It appears that MCC have increased access to medicinal cannabis treatment in Australia, however the concerns regarding care provided in these services [10, 11] highlight the importance of examining patient experience in different settings.

This project utilises data captured as part of the 2022 Cannabis As Medicine Survey (CAMS22) online consumer survey which included data on 2352 participants accessing medicinal cannabis treatment by prescription within the preceding 12 months [14]. The aim of this project was to compare the experiences of consumers accessing medicinal cannabis treatment from GHS to the experiences of those attending MCC, on the following:

- Participant characteristics: including demographics, history of cannabis use and self-reported health status;
- Current medicinal cannabis treatment: including type of health conditions treated, the quantity, route and cannabinoid composition of products used, and treatment costs;
- Consumer satisfaction with accessing medicinal cannabis treatment from their prescriber, including factors such as the amount of time spent in consultations, the amount of information provided to consumers, and integration with other health providers and/or interventions.

## Methods

CAMS-22 was an anonymous, cross-sectional, online survey of adult Australians (18 years or over) who had used cannabis for medical purposes in the previous 12 months. Details of the survey and study methods are described elsewhere [14]. The study was open from 16th of December 2022 to the 20th of April 2023 and was promoted through social media, consumer group webpages, and through medicinal cannabis treatment providers. Survey questions (see online materials for full questionnaire) included demographic characteristics, history of medical and non-medical cannabis use, health conditions treated with medical cannabis, physical and mental health status, and experiences with obtaining medicinal cannabis on prescription. Participants were asked to identify whether their “main prescriber of medicinal cannabis” worked in a “general health setting” or in a “cannabis clinic”. All participants provided informed consent, with no reimbursement for participation. The survey was approved by University of Sydney Human Research and Ethics Committee (HREC# 2022/433).

### Statistical analysis

The only covariate in all analyses was health setting, a two-level categorical variable indicating whether their main medicinal cannabis prescriber was employed in either a GHS or MCC group. To examine differences among respondents in these two groups we used single-level Bayesian regression models: standard linear regression for numeric outcomes, Bernoulli for binary categorical outcomes, multinomial logistic for categorical outcomes with three or more levels, and cumulative link models for ordinal variables. For the multinomial logistic models we used estimated marginal means to estimate the difference between the GHS and MCC groups in odds of belonging to each category of the outcome. All analyses were performed using R version 4.2.2, [16] specifically the brms [17] and emmeans [18] packages. All model parameters were estimated via four chains of 1000 samples each (4000 in total) with 1000 warm-up samples. Model diagnostics – $\:\stackrel{\text{-}}{\text{R}}$, traceplots and effective sample size (ESS) – were performed on all parameters. Priors were the default extremely broad non-informative priors supplied by the ‘brm()‘ function in the brms package: flat priors for betas, student’s t(3, 7, 2.5) for intercepts, and student’s t (3, 0, 2.5) for sigmas.

## Results

Demographic details and health status for the two groups are shown in Table 1. Most participants (1899/2394, 79.3%) accessed their medicinal cannabis mainly from MCC.

**Table 1:** Participant characteristics and health status based on treatment setting

					Comparison^a^ Estimate (95% CI)
	Type	Generalist Health Service	Medicinal Cannabis Clinic	Total	
Age, years, Range 18–87: M (SD)	Numeric	45.6 (13.8) *n*=495	42.1 (12.4) *n*=1899	42.8 (12.8) *N*=2394	**−3.5 (CI: −4.7, −2.2) **
Aboriginal or Torres Strait Islander: *n* (%)	Binary	24 (5%) *n*=495	77 (4%) *n*=1899	101 (4%) *N*=2394	0.8 (CI: 0.5, 1.3)
Education: n (%)	Categorical				
No further than secondary		96/495 (19%)	406/1895 (21%)	502/2390 (21%)	1.1 (CI: 0.9, 1.4)
Trade/Vocational qualification		199/495 (40%)	729/1895 (39%)	928/2390 (39%)	0.9 (CI: 0.8, 1.1)
University qualification		200/495 (40%)	760/1895 (40%)	960/2390 (40%)	1.0 (CI: 0.8, 1.2)
Employment: *n* (%)	Categorical				
Employed		253/474 (53%)	1298/1837 (71%)	1551/2311 (67%)	**2.1 (CI: 1.8, 2.5)**
Not employed		121/474 (26%)	278/1837 (15%)	399/2311 (17%)	**0.5 (CI: 0.4, 0.7)**
Disability		100/474 (21%)	261/1837 (14%)	361/2311 (16%)	**0.6 (CI: 0.5, 0.8)**
Days per week of work or study: n (%)	Numeric	2.4 (2.5), *n*=494	3.3 (2.4), *n*=1897	3.1 (2.5), *N*=2391	0.9 (CI: 0.6, 1.1)
Currently in a relationship: n (%)	Binary	314 (63%) *n*=495	1234 (65%) *n*=1899	1548 (65%) *N*=2394	1.1 (CI: 0.9, 1.3)
Sexual orientation: n (%)	Categorical				
Straight		386/495 (78%)	1441/1899 (76%)	1827/2394 (76%)	0.9 (CI: 0.7, 1.1)
Gay/Lesbian		24/495 (5%)	110/1899 (6%)	134/2394 (6%)	1.2 (CI: 0.8, 1.2)
Bisexual		50/495 (10%)	216/1899 (11%)	266/2394 (11%)	1.1 (CI: 0.9, 1.5)
Other		35/495 (7%)	132/1899 (7%)	167/2394 (7%)	1.0 (CI: 0.7, 1.4)
Days per week of tobacco: M (SD)	Numeric	1.2 (2.5) *n*=495	1.3 (2.6) *n*=1898	1.3 (2.6) *N*=2393	0.1 (−0.2, 0.4)
Days per week of alcohol: M (SD)	Numeric	1.0 (1.7), 0.3 (0, 1) *n*=495	1.2 (1.8) *n*=1898	1.2 (1.8) *N*=2393	**0.2 (CI: 0.0, 0.4)**
PROMIS-10: M (SD), IQR	Numeric	*N*=451	*N*=1750	*N*=2201	
Global Mental Health^b^		44.7 (9.8), 43.5 (38.8, 50.8)	47.0 (9.2), 48.3 (41.1, 53.3)	46.5 (9.4), 45.8 (41.1, 53.3)	**2.3 (CI: 1.3, 3.2)**
Global Physical Health^b^		43.6 (9.0), 47.7 (42.3, 54.1)	46.9 (8.3), 47.7 (42.3, 54.1)	46.2 (8.6), 47.7 (39.8, 50.8)	**3.2 (CI: 2.4, 4.2)**

Noteworthy differences in bold

^a^For numeric variables estimate is a mean difference, with noteworthy differences those whose confidence interval (CI) excludes 0. For binary or categorical variables estimate is an odds ratio, with noteworthy differences those whose CI excludes 1. MCC was reference group, therefore for numeric outcomes negative estimated mean difference (MCC – GHS) indicates mean in GHS group is higher. For categorical outcomes estimated odds ratios (MCC/GHS) below 1 indicate greater odds of event in question in GHS group. For ordinal outcomes estimated odds ratio (MCC/GHS) below one indicates greater odds of belonging to higher category in GHS group

^b^T-scores normed to a population mean of 50 and SD of 10

All parameters in all models had excellent diagnostics: ESS > 1000, $\:\stackrel{\text{-}}{\text{R}}$=1.00, and well-mixed, stationary traceplots. There were many notable differences between the GHS and MCC groups. People who obtained their medicinal cannabis prescription in a GHS were older, less likely to be employed, and self-rated their physical and mental health as significantly poorer than participants accessing treatment from MCC. There were no significant differences regarding gender, Indigenous status, education level or sexual orientation.

GHS participants (Table 2) were more likely to commence medical cannabis use at an older age, to never have used cannabis for non-medical reasons, and reported less concurrent non-medical cannabis use. GHS participants were more likely to be prescribed oral rather than inhaled products, and to use cannabis products with a higher proportion of cannabidiol than those in MCC group. A smaller proportion of GHS participants met Diagnostic and Statistical Manual of Mental Disorders version 5 (DSM5) criteria for moderate-severe cannabis use disorder [19] in the past 12 months (9% v 14%, OR = 1.6 (1.1, 2.2)). Whilst the average number of days per week of cannabis use was similar between the groups, the GHS group reported paying a mean of $18.10 (CI: 8.9, 28.1) less per week for their medication, representing approximately 15–20% less in cost than MCC participants.

**Table 2:** Cannabis use by treatment setting

	Type	Generalist Health Service	Medicinal Cannabis Clinic	Total	Comparison^a^ Estimate (95% CI)
Age first used cannabis (any reason): years, M (SD)	Numeric	26.7 (16.7), *n*=495	22.6 (12.8), *n*=1899	23.4 (13.8), *N*=2394	**−4.1 (CI: −5.5, −2.7)**
Age of first medical cannabis use, years: M (SD)	Numeric	39.7 (15.4), *n*=494	36.4 (13.7), *n*=1899	37.1 (14.2), *N*=2393	**−3.2 (CI: −4.5, −1.8)**
Age first nonmedical use^b^, years-old: M (SD)	Numeric	17.4 (5.9), *n*=326	17.3 (4.4), *n*=1466	17.3 (4.7), *N*=1792	−0.2 (CI: −0.7, 0.4)
Age first regular medical use, years-old: M (SD)	Numeric	40.1 (15.3) *n*=478	37.4 (13.4) *n*=1841	38.0 (13.9) *N*=2319	**−2.7 (CI: −4.1, 1.3)**
Never used cannabis for non-medical reason: n (%)	Binary	169/495 (34%)	433/1899 (23%)	602/2394 (25%)	**0.6 (CI: 0.5, 0.7)**
Current cannabis use only for medical reasons: n (%)	Binary	346/495 (70%)	1113/1899 (59%)	1459/2394 (61%)	**0.6 (CI: 0.5, 0.7)**
Percentage of cannabis use for medical purposes: Mean (SD), Median (IQR)	Numeric	93.9 (13.5), 100 (95.5, 100), *n*=495	90.6 (16.9), 100 (88, 100), *n*=1899	91.3 (16.3), 100 (90, 100), *N*=2394	**−3.3 (CI: −4.8, −1.7)**
Days per week cannabis use	Numeric	*n*=495	*n*=1899	*N*=2394	
Mean (SD), Median (IQR)					
Medical		6.0 (1.7), 7 (5.5, 7)	6.0 (1.7), 7 (5.5, 7)	6.0 (1.8), 7 (5.5, 7)	0.0 (CI: −0.2, 0.2)
Nonmedical		0.6 (1.6), 0 (0, 0)	0.7 (1.7), 0 (0, 0.25)	0.6 (1.7), 0 (0, 0)	0.1 (CI: −0.1, 0.3)
Any		6.0 (1.8), 7 (5.9, 7)	6.0 (1.8), 7 (5.5, 7)	6.0 (1.8), 7 (5.8, 7)	−0.0 (CI: −0.2, 0.2)
Route of Administration: n (%)	Categorical				
Vaporised		157/483 (33%)	873/1860 (47%)	1030/2343 (44%)	**1.8 (CI: 1.5, 2.2)**
Oral		226/483 (47%)	516/1860 (28%)	742/2343 (32%)	**0.4 (CI: 0.4, 0.5)**
Smoked		100/483 (21%)	471/1860 (25%)	571/2343 (24%)	**1.3 (CI: 1.1, 1.6)**
Composition of medical cannabis^c^: n (%)	Ordinal				**0.6 (CI: 0.5, 0.8)**
THC only		75/468 (16%)	335/1833 (18%)	410/2301 (18%)	
Mostly THC		174/468 (37%)	856/1833 (47%)	1030/2301 (45%)	
Equal THC/CBD		121/468 (26%)	430/1833 (24%)	551/2301 (24%)	
Mostly CBD		43/468 (9%)	130/1833 (7%)	173/2301 (8%)	
CBD only		55/468 (12%)	82/1833 (4%)	137/2301 (6%)	
Cost of medical cannabis: Mean (SD), Median (IQR)	Numeric	*n*=494	*n*=1898	2392	
$AUD per week					
Medication		87.6 (87.8), 55 (30,100)	106 (102), 75 (40,150)	102 (99.2), 75 (30, 110)	**18.1 (CI: 8.9, 28.1)**
Other costs		34.0 (54.8), 15 (5, 40)	35.1 (61.4), 20 (8, 30)	34.9 (60.1), 19 (6, 35)	**1.1 (CI: −4.9, 7.1)**
Any CUD (≥2/11 criteria met): n (%)	Binary	141/470 (30%)	698/1812 (39%)	839/2282 (37%)	**1.5 (CI: 1.2, 1.8)**
Moderate-Severe CUD (≥4/11 criteria met): n (%)	Binary	44/470 (9%)	255/1812 (14%)	299/2282 (13%)	**1.6 (CI: 1.1, 2.2)**

^a^See legend Table 1

^b^Of the 2394 respondents included in this analysis, 602 indicated that they had never used cannabis nonmedically, leaving 1792 to answer the questions concerning nonmedical (or any) use

^c^Ordered by proportion of CBD (THC only < CBD only)

GHS participants were more likely to use medicinal cannabis to treat a pain condition and less likely to be treating a mental health condition such as anxiety, depression or post-traumatic stress disorder (Table 3). A marked difference between the two groups was whether the participants’ prescriber also attended to other healthcare issues – with 49% of participants in the GHS group reporting their prescribing doctor also treated other health conditions, compared to 3% in the MCC group (OR = 0.03, 95%CI: 0.02, 0.04).

**Table 3:** Health conditions treated

	Type	Generalist Health Service	Medicinal Cannabis Clinic	Total	Comparison^a^ Estimate (95% CI)
Main condition: general category: n (%)	Categorical				
Mental Health		128/491 (26%)	686/1883 (36%)	814/2374 (34%)	**1.6 (CI: 1.3, 2.0)**
Pain		225/491 (46%)	677/1883 (36%)	902/2374 (16%)	**0.7 (CI: 0.6, 0.8)**
Sleep		71/491 (15%)	315/1883 (17%)	386/2374 (16%)	1.2 (CI: 0.9, 1.5)
Neurological		28/491 (6%)	80/1883 (4%)	108/2374 (5%)	0.7 (CI: 0.5, 1.1)
Other		39/491 (8%)	125/1883 (7%)	164/2374 (7%)	0.8 (CI: 0.6, 1.2)
Main condition: top 6 conditions: n (%)	Categorical				
Anxiety		74/491 (15%)	402/1883 (21%)	476/2374 (20%)	**1.5 (CI: 1.2, 2.0)**
Insomnia		61/491 (12%)	264/1883 (14%)	325/2374 (14%)	1.2 (CI: 0.9, 1.5)
Back pain		64/491 (13%)	255/1883 (14%)	319/2374 (13%)	1.1 (CI: 0.8, 1.4)
Arthritis		44/491 (9%)	120/1883 (6%)	164/2374 (7%)	**0.7 (CI: 0.5, 1.0)** ^b^
Post Traumatic Stress Disorder		16/491 (3%)	91/1883 (5%)	107/2374 (5%)	**1.6 (CI: 1.0, 2.5)**
Depression		11/491 (2%)	93/1883 (5%)	104/2374 (4%)	**2.3 (CI: 1.4, 4.1)**
Global care provided by prescriber: n (%)	Categorical				
Doctor also treats other health issues		243/495 (49%)	51/1898 (3%)	293/2393 (12%)	**0.03 (CI: 0.02, 0.04)**
Doctor only treats medical cannabis conditions		230/495 (47%)	1745/1898 (92%)	1975/2393 (83%)	**13.2 (CI: 10.1, 16.2)**
I have no other health issues		22/495 (4%)	102/1898 (5.4%)	124 (5%)	1.2 (CI: 0.9, 1.9)

^a^See legend Table 1

^b^Upper bound of 95% CI less than 1.0 but, with one decimal point, rounds up to 1.0

Whilst there were generally high levels of satisfaction by participants in both groups regarding most aspects of care (Table 4), there were higher levels of satisfaction amongst GHS participants regarding all the satisfaction items in the survey – including the amount of time spent in consultations (OR = 0.8 (0.7–0.9) for initial consultation and OR = 0.7 (0.6, 0.9) for follow-up appointments), the amount of information provided ((OR = 0.7 (0.6, 0.9) for harms and benefits, and OR = 0.7 (0.6, 0.8) for evidence)), treatment fees (OR = 0.6, (0.5, 0.7)), and the use of other health care approaches (e.g. other medications, physical therapies, counselling) to treat their condition (OR = 0.5, (0.4, 0.6)).

**Table 4:** Participant levels of satisfaction with health care provided, by treatment setting

		Generalist Health Service (*n* = 495)	Medicinal Cannabis Clinic (*n* = 1898)	Total (*N* = 2393)	Odds Ratio^a^ (95% CI)
Amount of information about harms and benefits of medical cannabis	Very satisfied Satisfied Neutral Dissatisfied Very dissatisfied	32 (65%) 114 (23%) 38 (8%) 17 (3%) 4 (1%)	1043 (55%) 578 (30%) 194 (10%) 61 (3%) 22 (1%)	1365 (57%) 692 (29%) 232 (10%) 78 (3%) 26 (1%)	**0.7 (CI: 0.6**,** 0.9)**
Amount of information about evidence of medical cannabis for health condition	Very satisfied Satisfied Neutral Dissatisfied Very dissatisfied	287 (58%) 125 (25%) 60 (12%) 18 (4%) 5 (1%)	900 (47%) 624 (33%) 281 (15%) 64 (3%) 29 (2%)	1187 (50%) 749 (31%) 341 (14%) 82 (3%) 34 (1%)	**0.7 (CI: 0.6**,** 0.8)**
Amount of time spent in initial assessment	Very satisfied Satisfied Neutral Dissatisfied Very dissatisfied	308 (62%) 132 (27%) 34 (7%) 13 (3%) 8 (2%)	1041 (54%) 591 (31%) 180 (9%) 60 (3%) 26 (1%)	1349 (56%) 723 (30%) 214 (9%) 73 (3%) 34 (1%)	**0.8 (CI: 0.7**,** 0.9)**
Amount of time spent follow-up appointments	Very satisfied Satisfied Neutral Dissatisfied Very dissatisfied	278 (56%) 133 (27%) 57 (12%) 18 (4%) 9 (2%)	882 (47%) 571 (30%) 323 (17%) 93 (5%) 29 (2%)	1160 (49%) 704 (29%) 380 (16%) 111 (5%) 38 (2%)	**0.7 (CI: 0.6**,** 0.9)**
Doctor addresses questions or concerns	Very satisfied Satisfied Neutral Dissatisfied Very dissatisfied	323 (65%) 116 (23%) 41 (8%) 11 (2%) 4 (1%)	1094 (58%) 560 (30%) 175 (9%) 42 (2%) 27 (1%)	1417 (59%) 676 (28%) 216 (9%) 53 (2%) 31 (1%)	**0.8 (CI: 0.6**,** 0.9)**
Doctor uses approaches other than medical cannabis to treat medical condition	Very satisfied Satisfied Neutral Dissatisfied Very dissatisfied	273 (55%) 121 (24%) 80 (16%) 15 (3%) 6 (1%)	699 (37%) 479 (25%) 593 (31%) 84 (4%) 43 (2%)	972 (41%) 600 (25%) 673 (28%) 99 (4%) 49 (2%)	**0.5 (CI: 0.4**,** 0.6)**
Fees: n (%)	Very satisfied Satisfied Neutral Dissatisfied Very dissatisfied	148 (30%) 97 (20%) 145 (29%) 62 (13%) 43 (9%)	303 (16%) 411 (22%) 635 (34%) 371 (20%) 178 (9%)	451 (20%) 508 (21%) 780 (32%) 433 (18%) 221 (9%)	**0.6 (CI: 0.5**,** 0.7)**

Noteworthy differences in bold

^a^Estimated odds ratio (MCC/GHS) below one indicates greater odds of belonging to higher category in GH group

## Discussion

This study identified significant differences in the characteristics of people accessing medicinal cannabis treatment in MCC and GHS. Compared to GHS, MCC participants were younger and had higher rates of employment, were less likely to be prescribed oral products and less likely to use cannabidiol (CBD) containing products. MCC participants were more likely to access treatment for mental health and less likely for chronic pain conditions. MCC participants had lower satisfaction levels regarding consultation duration, information about potential harms and benefits, use of other treatment approaches and treatment costs.

The emergence of MCC has been a key factor in the marked increase in the number of patients prescribed medicinal cannabis in Australia. However, to date there has been little research examining their impact upon patient experience, access, quality of health care and outcomes. MCC represent a somewhat different business model to the organisation of normal health care for patients – essentially clinics that prioritise the use of a class of unapproved medications for a diverse range of patients and health conditions – from childhood epilepsy to palliative care in elderly patients. Proponents of these clinic models can rightfully point to their important role in increasing patient access to medicinal cannabis, particularly given the reluctance of many doctors to prescribe cannabis medicines. The normalisation of telehealth medicine in recent years has also facilitated the business models of these clinics, providing access to patients to clinics across Australia without the need for face-to-face consultations. It is unknown what proportion of patients across Australia access medicinal cannabis through MCC. In this study, almost 80% of participants were mainly accessing their medicinal cannabis through such clinics, although this may reflect sampling bias of our study, with 18% of CAMS-22 respondents recruited through advertisements in MCC [14]. Notwithstanding, our findings suggest MCC are a major provider of medicinal cannabis treatment in Australia, with possibly hundreds of thousands of Australians using them each year.

Commentators have raised concerns regarding the fragmentation of health care and/or over-reliance of a single treatment approach for managing what are often complex and chronic health conditions that usually benefit from multimodal care (e.g. chronic pain, mental health) [10, 11]. Our findings indeed suggest greater fragmentation of healthcare in these clinics: few (3%) participants reported that their MCC doctor also attended to other health problems, compared to approximately half (49%) in GHS – which is to be expected given the business models of MCC to focus on health conditions treated with medicinal cannabis. GHS participants reported higher levels of satisfaction regarding the extent to which other interventions (e.g. other medications, counselling, physiotherapy) were incorporated into the treatment of their health condition by their medicinal cannabis prescriber. The majority of patients in Australia access medicinal cannabis for the treatment of chronic health conditions [2] (e.g. chronic pain, mental health), which often benefit from the integration of multiple different treatment approaches (e.g. counselling, medications, physical therapies, peer support) [20], usually from a range of healthcare providers. The tendency towards the compartmentalisation of healthcare with medicinal cannabis treatment is a concern, requiring greater attention to effective communication and coordination across healthcare providers, particularly when prescribing psychoactive medications such as tetrahydrocannabinol (THC).

Another concern identified regarding MCC is the potential for higher patient fees for accessing medicinal cannabis treatment, and implications for patient access and equity. There were significantly lower levels of satisfaction amongst MCC patients regarding overall costs of medicinal cannabis treatment, and our data suggests that MCC patients were spending almost 20% more on treatment costs. It remains unclear whether the greater medication costs reflect the use of higher doses and/or more expensive products amongst the MCC patients – which could possibly be a consequence of vertical integration of some MCC. Alternatively this could reflect higher consultation fees, and/or reflect a greater ‘capacity to pay’ given that MCC patients were more likely to be employed (and probably have greater income) than those attending generalist settings.

Another critique of MCC is that they service people seeking ‘legal’ cannabis for non-medical reasons, and that prescribers are engaging in “pseudo-recreational or unsound medical practices” (p.1286) [10]. Our data indeed do suggest differences in the profile of patients attending the different settings – those attending MCC were more likely to be younger, to have a longer history of non-medical cannabis use, to use higher THC-containing products, to use a higher proportion of cannabis for non-medical reasons, and to meet criteria for a Cannabis Use Disorder. However, whilst there are statistical differences between treatment settings on these variables, it must be emphasised that only a minority of participants from both settings met criteria for Cannabis Use Disorder and non-medical use of cannabis was reported by a minority of participants across the study. This suggests that broad generalizations that MCC are ‘cannabis distribution centres’ rather than legitimate healthcare services appear largely unfounded, and potentially increase stigmatization of patients and healthcare providers in these settings.

Our study also aimed to examine the levels of satisfaction amongst patients regarding different aspects of healthcare associated with their treatment. Overall there were high levels of satisfaction amongst both groups of patients regarding the amount of information provided (potential benefits, side effects, evidence of efficacy, willingness to address questions) and in the amount of time spent in initial and follow-up consultations – although satisfaction levels were significantly higher on these issues amongst GHS patients. It should be emphasised however, that consumer satisfaction is only one aspect of assessing the appropriateness and quality of health care. Some patients may be satisfied with brief consultations or compartmentalising of their healthcare across different treatment providers – but that does not necessarily equate with high quality health care [11]. Further research is required examining the appropriateness of treatment with medicinal cannabis (especially as these are unapproved medicines by the Therapeutics Goods Administration), and of patient outcomes beyond patient satisfaction. One Australian longitudinal cohort study [21] suggested positive outcomes and few safety concerns in 3961 patients treated in a particular MCC for predominately chronic pain and psychiatric indications, although the study was not independent of the clinics providing treatment, and independent evaluations are needed. We should also be cautious in categorising the quality of care provided solely on the basis of the setting in which it is delivered, at risk of stigmatising both patients and service providers involved in MCC – indeed some participants reported either ‘very high’ and ‘very low’ levels of satisfaction across both health settings in our survey.

Our study has a number of limitations – it involves self-reported online data from anonymous individuals – and as such we cannot validate responses. A possible limitation is whether some participants may not have been aware whether their prescribing doctor worked in a MCC or GHS. The study also may suffer from sampling bias limiting the extent to which the findings can be generalised. For example, individuals who are satisfied and remain in medicinal cannabis treatment may have been more likely to participate in the survey than dissatisfied individuals who discontinued treatment.

In conclusion, whilst the experiences of consumers in this survey suggest generally positive levels of satisfaction with medicinal cannabis treatment delivered across both MCC and GHS settings, there were considerably higher levels of satisfaction amongst consumers accessing treatment in generalist settings. Although MCC appear to provide easier access for patients, our findings suggest potential concerns regarding the fragmentation of health care of complex chronic health conditions in these settings. Further research is required to examine other dimensions of quality and appropriateness of these clinic models.

## Supplementary Information

Supplementary Material 1.

## Data Availability

All data and code used to perform analyses are publicly available at https://osf.io/3q4h8/.

## References

[1] Office of Drug Control. Medicinal cannabis. Australia: Commonwealth of Australia. https://www.odc.gov.au/medicinal-cannabis (viewed 17th January 2025).

[2] Therapeutics Goods Authority. Medicinal Cannabis: Access Pathways and Patient Access. https://www.tga.gov.au/products/unapproved-therapeutic-goods/medicinal-cannabis-hub/medicinal-cannabis-access-pathways-and-patient-access-data. (viewed 17th January 2025).

[3] Therapeutic Goods Administration. Special Access Scheme (SAS): Guidance for health practitioners accessing unapproved therapeutic goods. https://www.tga.gov.au/resources/guidance/special-access-scheme-sas-guidance-health-practitioners-accessing-unapproved-therapeutic-goods (viewed 14 August 2025).

[4] MacPhail SL, Bedoya-Perez MA, Cohen R, Kotsirilos V, McGregor IS, Cairns EA. Medicinal cannabis prescribing in australia: an analysis of trends over the first five years. Front Pharmacol. 2022;13:885655. 10.3389/fphar.2022.885655. 35620292 10.3389/fphar.2022.885655 PMC9127064

[5] Community Affairs References Committee. Current barriers to patient access to medicinal cannabis in Australia [Internet]. Canberra: Commonwealth of Australia; 2020. https://www.aph.gov.au/Parliamentary_Business/Committees/Senate/Community_Affairs/Medicinalcannabis/Report. (viewed 17th January 2025).

[6] Lintzeris N, Mills L, Suraev A, et al. Medical cannabis use in the Australian community following introduction of legal access: the 2018–2019 online Cross-Sectional cannabis as medicine survey (CAMS-18). Harm Reduct J. 2020;17(1). 10.1186/s12954-020-00377-0. 10.1186/s12954-020-00377-0 PMC7278204 32513180

[7] Lintzeris N, Mills L, Abelev SV, Suraev A, Arnold JC, McGregor IS. Medical cannabis use in australia: consumer experiences from the online cannabis as medicine survey 2020 (CAMS-20). Harm Reduct J. 2022;19(1):1–88. 10.1186/s12954-022-00666-wCAMS18. 35907959 10.1186/s12954-022-00666-w PMC9338505

[8] Gardiner KM, Singleton JA, Sheridan J, et al. Health professional beliefs, knowledge and concerns surronding medicinal cannabis – A systematic review. PLoS ONE. 2019;14:e0216556. 31059531 10.1371/journal.pone.0216556 PMC6502454

[9] O’Rourke R, Lima ML, Jetten J. Healthcare professionals and medical cannabis: a scoping review informed by theoretical domains framework. J Public Health (Berl). 2022;30:1901–13.

[10] Dobson O, Barber M, Graham M, Carter A, Savic M. The wild West of medicine’: A qualitative investigation of the factors influencing Australian health-care practitioners’ delivery of medicinal cannabis. Drug Alcohol Rev. 2024;43(5):1280–93. 38630896 10.1111/dar.13847

[11] Martin JH, Hall W, Fitzcharles MA, Borgelt L, Crippa J. Ensuring access to safe, effective, and affordable cannabis-based medicines. Br J Clin Pharmacol. 2020;86:630–4. 32128867 10.1111/bcp.14242 PMC7098857

[12] Snoswell CL, Caffrey LJ, Haydon HM, Thomas EE, Smith AC. Telehealth uptake in general practice as a result of the coronavirus (COVID-19) pandemic. Aust Health Rev. 44;(5);737–40. 10.1071/AH20183. PMID: 32853536. 10.1071/AH20183 32853536

[13] Penington Institute. 2024. Cannabis in Australia 2024.Melbourne: Penington Institute. ISSN: 2653–7087. https://www.penington.org.au/cannabis-in-australia-report-2024/ (viewed 12 December 2024).

[14] Mills L, Arnold JC, Suraev A, et al. Medical cannabis use in Australia seven years after legalisation: findings from the online cannabis as medicine survey 2022–2023 (CAMS-22). Harm Reduct J. 2024;21(1):104–104. 10.1186/s12954-024-00992-1. 38807133 10.1186/s12954-024-00992-1 PMC11131250

[15] Medical Cannabis Users Association of Australia. Submission 9 to Senate community affairs references committee inquiry. Current barriers to patient access to medicinal cannabis in Australia. 2020. https://parlinfo.aph.gov.au/parlInfo/download/committees/reportsen/024403/toc_pdf/.

[16] R: A Language and environment for statistical computing. R Foundation for Statistical Computing; 2016. https://www.r-project.org/

[17] Bürkner P-C. Brms: an R package for bayesian multilevel models using Stan. J Stat Softw. 2017;80(1):1–28.

[18] Lenth RV. emmeans: Estimated Marginal Means, aka Least-Squares Means. R package Version 1-5-3. 2020. https://rvlenth.r-universe.dev/emmeans. Accessed 29 Sep 2025.

[19] American Psychiatric Association DSMTF, American Psychiatric A. Diagnostic and statistical manual of mental disorders: DSM-5. Arlington, Va: American Psychiatric Association; 2013.

[20] Dale R, Stacey B. Multimodal treatment of chronic pain. Med Clin North Am. 2016;100(1):55–64. 10.1016/j.mcna.2015.08.012. 26614719 10.1016/j.mcna.2015.08.012

[21] Vickery AW, Roth S, Ernenwein T, Kennedy J, Washer P. A large Australian longitudinal cohort registry demonstrates sustained safety and efficacy of oral medicinal cannabis for at least two years. PLoS ONE. 2022;17(11):e0272241. 36399463 10.1371/journal.pone.0272241 PMC9674134

