# Supplementary material for: Consumer perspectives of accessing medicinal cannabis treatment from cannabis clinics versus generalist health settings in Australia
Source: J Cannabis Res. 2025 Oct 27;7:83. doi: 10.1186/s42238-025-00338-z (PMC12557907; doi:10.1186/s42238-025-00338-z)
Supplement: Supplementary file 1 — Supplementary Material 1. [file 42238_2025_338_MOESM1_ESM.pdf]

Online Supplementary materials

Full Cams-22 Questionnaire

The Cannabis As Medicine Survey: 2022

Please complete the survey below.

Progress bars will appear throughout the survey showing you how far through you are. Please note that you can save your progress at any time and return to complete the survey later. To do so you must record the User ID code provided when you select the "save and return" option at the end of any page.

Thank you! We really appreciate you sharing your experiences.

As researchers from the University of Sydney we are launching an Australia-wide online survey of people who have used cannabis for MEDICAL (therapeutic) purposes within the last 12 months. Similar surveys were conducted in 2016, 2018, and 2020, and we aim to see how things have changed since the last survey.

The survey will be anonymous and confidential: There is no way your responses can be matched to personal and/or identifying data.

The survey asks about:

1. You: your age, gender, level of education and current living and work situations

2. Conditions you have used medical cannabis to treat in the last 12 months

3. How you access and administer cannabis for medical purposes: LEGAL (PRESCRIBED) OR ILLICIT (NON-PRESCRIBED). These categories are in line with Federal legislation.

4. The extent to which you experience benefits (e.g. symptom relief) and/or problems (e.g. side effects, social or legal problems) from your use of medical cannabis.

5. How you would like cannabis products to be available for medical reasons in the future

6. Your general health and recent use of other substances.

7. How medical cannabis may have affected your driving behaviour.

PLEASE NOTE OUR DEFINITION OF MEDICAL CANNABIS: Medical Cannabis refers to any legal or illegal product (oil, capsule, spray, plant material etc) derived from cannabis or hemp. This includes products containing only THC, or only CBD, or any mixture of CBD and THC, or products where the THC/CBD content is not known.

For further details and instructions please read the participant information statement by clicking on the link below.

Please indicate your eligibility for the study below:

○ I am an Australian resident over the age of 18 who has used cannabis for MEDICAL reasons in the last 12 MONTHS. I understand the definition of medical cannabis above. Click here to confirm.

Informed consent

______

[Attachment: "ParticipantInformationStatement_CAMS22.pdf"]

Survey Progress
1%

SECTION 1: DEMOGRAPHIC INFORMATION

In this section we will ask you questions about your basic demographic information and where you heard about the survey.

1.01 Please tell us your age in years

____
(Years old)

1.02 Which state/territory do you live in?

- ◯ New South Wales
- ◯ Victoria
- ◯ Queensland
- ◯ Northern Territory
- ◯ Western Australia
- ◯ South Australia
- ◯ Tasmania
- ◯ Australian Capital Territory

1.03 What is the post code of your current address?

____

1.04 Are you of Aboriginal or Torres Strait Islander origin?

- ◯ No
- ◯ Yes, Aboriginal
- ◯ Yes, Torres Strait Islander
- ◯ Yes, both Aboriginal and Torres Strait Islander

1.05 Do you speak a language other than English at home?

- ◯ No, I only speak English at home
- ◯ Yes, I speak a language other than English at home

1.05a Please tell us us what other language you speak at home

____

1.06 What is the highest level of education you have completed?

- ◯ Primary School
- ◯ Secondary/High school
- ◯ Trade or Vocational training (e.g. TAFE or college)
- ◯ Undergraduate University degree (e.g. Bachelors, Honours)
- ◯ Postgraduate University degree (e.g. Masters, PhD)
- ◯ Other

1.06a How else would you describe your highest level of education?

____
(please describe)

1.07 Which option best describes your current employment status?

- ◯ Full time work
- ◯ Part time work
- ◯ Home duties
- ◯ Unemployed
- ◯ Retired
- ◯ Disability pension
- ◯ Student
- ◯ Other

1.07a Please describe the type of employment you are currently engaged in

____

1.07b Are you receiving pension or unemployment benefits?

- ◯ Yes
- ◯ No

1.07c Did your employment status change as a result of the COVID-19 pandemic?

- ◯ Yes
- ◯ No

1.07d Which option best describes your employment status prior to the COVID-19 pandemic?

◯ Full time work
◯ Part time work
◯ Home duties
◯ Unemployed
◯ Retired
◯ Disability pension
◯ Student
◯ Other

1.07da How would you best describe your employment status prior to the pandemic?

____

1.08 Which of the following best describes the sector you have been employed in for the majority of your working life?

◯ Hospitality
◯ Retail
◯ Office
◯ Healthcare
◯ Construction
◯ Education
◯ Manufacturing
◯ Transport
◯ Mining
◯ Law enforcement
◯ Entertainment
◯ Never employed
◯ Other

1.08a How would you best describe the sector you have been employed in for the majority of your working life?

____

1.09 On how many days of the last 28 days did you engage in PAID work and/or study?

Please note that paid work does not include voluntary work.

Study includes school, TAFE, university or vocational training.

____
(Please enter a value from 0-28 (Full time = 20 days))

1.1 What is your current relationship status?

◯ Single (not currently in a relationship)
◯ Partnered (currently in a relationship)

1.11 What was your sex recorded at birth?

◯ Male
◯ Female

1.12 What is your gender?

◯ Male
◯ Female
◯ Non-binary
◯ Different term
◯ Prefer not to answer

1.12a Please tell us which gender identity you most readily identify as.

____
(please specify)

1.13 Please tell us your sexual orientation

- ◯ Straight (heterosexual)
- ◯ Gay or lesbian
- ◯ Bisexual
- ◯ Different term
- ◯ Don't know
- ◯ Prefer not to answer

1.13a Please tell us how you would describe your sexual orientation

____

1.14 Where did you first hear about this Survey?

- ◯ Facebook
- ◯ Other social media (e.g. Instagram, Twitter, Snapchat)
- ◯ Online forum (e.g. Reddit, Whirlpool, Bluelight)
- ◯ A friend
- ◯ Medical cannabis provider
- ◯ Consumer support group
- ◯ Lambert Initiative website
- ◯ Doctor/healthcare provider
- ◯ Private cannabis clinic (e.g. CA clinic, Tetra health, etc.)
- ◯ Media (newspaper, radio, TV)
- ◯ Other

(select one option)

1.14a From what other source did you first hear about this survey?

____
(please describe)

Survey Progress
5%

SECTION 2

TREATMENT WITH MEDICAL CANNABIS

Please provide us with some information about what medical conditions you treat with cannabis

2.01 Have you used medical cannabis to treat a condition from any of the following groups?

You may select more than one option

- ☐ Cancer
- ☐ Gastro-intestinal
- ☐ Mental health and/or substance use
- ☐ Neurological
- ☐ Pain
- ☐ Sleep
- ☐ Other

(select all that apply)

2.01a Please indicate which of the following cancer conditions you have used medical cannabis to treat.

- ☐ Blood cancers (leukaemia, lymphoma, myeloma)
- ☐ Brain cancers (glioblastoma, neuroblastoma, mesothelioma)
- ☐ Breast cancer
- ☐ Gastrointestinal cancer (bowel, colon, stomach, pancreatic)
- ☐ Lung cancer
- ☐ Reproductive cancers (prostate, cervical, uterine, testicular, ovarian)
- ☐ Skin cancers (melanoma)
- ☐ Other forms of cancer

(select all that apply (limit of three))

2.01a What other type of cancer have you used medical cannabis to treat? __________________________________

2.01b Have you been diagnosed with blood cancer or had it confirmed in some way by a healthcare provider?
- ◯ Yes
- ◯ No

2.01b Have you been diagnosed with brain cancer or had it confirmed in some way by a healthcare provider?
- ◯ Yes
- ◯ No

2.01b Have you been diagnosed with breast cancer or had it confirmed in some way by a healthcare provider?
- ◯ Yes
- ◯ No

2.01b Have you been diagnosed with gastrointestinal cancer or had it confirmed in some way by a healthcare provider?
- ◯ Yes
- ◯ No

2.01b Have you been diagnosed with lung cancer or had it confirmed in some way by a healthcare provider?
- ◯ Yes
- ◯ No

2.01b Have you been diagnosed with reproductive cancer or had it confirmed in some way by a healthcare provider?
- ◯ Yes
- ◯ No

2.01b Have you been diagnosed with skin cancer or had it confirmed in some way by a healthcare provider?
- ◯ Yes
- ◯ No

2.01b Have you been diagnosed with [q02_01a_condition_cancer_other] or had it confirmed in some way by a healthcare provider?
- ◯ Yes
- ◯ No

2.01b Please check the option that best describes how your blood cancer is now, compared with how it was before using medical cannabis.
- ◯ Very much better
- ◯ Much Better
- ◯ A little better
- ◯ No change
- ◯ A little worse
- ◯ Much worse
- ◯ Very much worse

2.01b Please check the option that best describes how your brain cancer is now, compared with how it was before using medical cannabis.
- ◯ Very much better
- ◯ Much Better
- ◯ A little better
- ◯ No change
- ◯ A little worse
- ◯ Much worse
- ◯ Very much worse

2.01b Please check the option that best describes how your breast cancer is now, compared with how it was before using medical cannabis.
- ◯ Very much better
- ◯ Much Better
- ◯ A little better
- ◯ No change
- ◯ A little worse
- ◯ Much worse
- ◯ Very much worse

2.01b Please check the option that best describes how your gastrointestinal cancer is now, compared with how it was before using medical cannabis.

◯ Very much better
◯ Much Better
◯ A little better
◯ No change
◯ A little worse
◯ Much worse
◯ Very much worse

2.01b Please check the option that best describes how your lung cancer is now, compared with how it was before using medical cannabis.

◯ Very much better
◯ Much Better
◯ A little better
◯ No change
◯ A little worse
◯ Much worse
◯ Very much worse

2.01b Please check the option that best describes how your reproductive cancer is now, compared with how it was before using medical cannabis.

◯ Very much better
◯ Much Better
◯ A little better
◯ No change
◯ A little worse
◯ Much worse
◯ Very much worse

2.01b Please check the option that best describes how your skin cancer is now, compared with how it was before using medical cannabis.

◯ Very much better
◯ Much Better
◯ A little better
◯ No change
◯ A little worse
◯ Much worse
◯ Very much worse

2.01b Please check the option that best describes how your [q02_01a_condition_cancer_other] is now, compared with how it was before using medical cannabis.

◯ Very much better
◯ Much Better
◯ A little better
◯ No change
◯ A little worse
◯ Much worse
◯ Very much worse

2.01a Please indicate which of the following gastro-intestinal conditions you have used medical cannabis to treat.

☐ Crohn's disease
☐ Irritable bowel syndrome
☐ Ulcerative collitis
☐ Other gastro-intestinal condition
(select all that apply (limit of three))

2.01a What other type of gastro-intestinal conditions have you used medical cannabis to treat?

____

2.01b Have you been diagnosed with Crohn's disease or had it confirmed in some way by a healthcare provider?

◯ Yes
◯ No

2.01b Have you been diagnosed with Irritable Bowel Syndrome or had it confirmed in some way by a healthcare provider?

◯ Yes
◯ No

2.01b Have you been diagnosed with Ulcerative Colitis or had it confirmed in some way by a healthcare provider?

◯ Yes
◯ No

	Have you been diagnosed with [q02_01a_condition_gastro_other] or had it confirmed in some way by a healthcare provider?	◯ Yes<br>◯ No
2.01b	Please check the option that best describes how your Crohn's disease is now, compared with how it was before using medical cannabis.	◯ Very much better<br>◯ Much Better<br>◯ A little better<br>◯ No change<br>◯ A little worse<br>◯ Much worse<br>◯ Very much worse
2.01b	Please check the option that best describes how your Irritable Bowel Syndrome is now, compared with how it was before using medical cannabis.	◯ Very much better<br>◯ Much Better<br>◯ A little better<br>◯ No change<br>◯ A little worse<br>◯ Much worse<br>◯ Very much worse
2.01b	Please check the option that best describes how your Ulcerative Colitis is now, compared with how it was before using medical cannabis.	◯ Very much better<br>◯ Much Better<br>◯ A little better<br>◯ No change<br>◯ A little worse<br>◯ Much worse<br>◯ Very much worse
2.01b	Please check the option that best describes how your [q02_01a_condition_gastro_other] is now, compared with how it was before using medical cannabis.	◯ Very much better<br>◯ Much Better<br>◯ A little better<br>◯ No change<br>◯ A little worse<br>◯ Much worse<br>◯ Very much worse
2.01a	Please indicate which of the following mental health conditions you have used medical cannabis to treat.	☐ Addiction (Cannabis)<br>☐ Addiction (Other drugs: including alcohol, opioid, amphetamine)<br>☐ Anxiety Disorder (e.g. generalised anxiety, panic disorder, OCD)<br>☐ Attention Deficit disorder (ADHD)<br>☐ Depression<br>☐ Eating disorders (e.g. anorexia / bulimia/ obesity)<br>☐ Manic Disorder (Bipolar affective disorder)<br>☐ Post-Traumatic Stress Disorder<br>☐ Schizophrenia or other psychosis<br>☐ Other mental health condition<br>(select all that apply (limit of three))
2.01a	What other type of mental health condition do you use prescribed medical cannabis to treat?	________________________
2.01b	Have you been diagnosed with cannabis dependence or cannabis use disorder or had it confirmed in some way by a healthcare provider?	◯ Yes<br>◯ No

2.01b	Have you been diagnosed with dependence on a substance other than cannabis or substance use disorder other than cannabis use disorder, or had it confirmed in some way by a healthcare provider?	◯ Yes<br>◯ No
2.01b	Have you been diagnosed with an Anxiety disorder or had it confirmed in some way by a healthcare provider?	◯ Yes<br>◯ No
	Have you been diagnosed with an Attention Deficit disorder or had it confirmed in some way by a healthcare provider?	◯ Yes<br>◯ No
2.01b	Have you been diagnosed with a depressive disorder or had it confirmed in some way by a healthcare provider?	◯ Yes<br>◯ No
2.01b	Have you been diagnosed with an eating disorder or had it confirmed in some way by a healthcare provider?	◯ Yes<br>◯ No
2.01b	Have you been diagnosed with a manic disorder or had it confirmed in some way by a healthcare provider?	◯ Yes<br>◯ No
2.01b	Have you been diagnosed with Post-Traumatic Stress Disorder (PTSD) or had it confirmed in some way by a healthcare provider?	◯ Yes<br>◯ No
2.01b	Have you been diagnosed with schizophrenia or other psychosis or had it confirmed in some way by a healthcare provider?	◯ Yes<br>◯ No
2.01b	Have you been diagnosed with [q02_01a_condition_mentalh_other] or had it confirmed in some way by a healthcare provider?	◯ Yes<br>◯ No
2.01b	Please check the option that best describes how your cannabis dependence or cannabis use disorder is now, compared with how it was before using medical cannabis.	◯ Very much better<br>◯ Much Better<br>◯ A little better<br>◯ No change<br>◯ A little worse<br>◯ Much worse<br>◯ Very much worse
2.01b	Please check the option that best describes how your non-cannabis substance dependence or non-cannabis substance use disorder is now, compared with how it was before using medical cannabis.	◯ Very much better<br>◯ Much Better<br>◯ A little better<br>◯ No change<br>◯ A little worse<br>◯ Much worse<br>◯ Very much worse
2.01b	Please check the option that best describes how your Anxiety disorder is now, compared with how it was before using medical cannabis.	◯ Very much better<br>◯ Much Better<br>◯ A little better<br>◯ No change<br>◯ A little worse<br>◯ Much worse<br>◯ Very much worse

2.01b Please check the option that best describes how your Attention Deficit disorder is now, compared with how it was before using medical cannabis.

- ◯ Very much better
- ◯ Much Better
- ◯ A little better
- ◯ No change
- ◯ A little worse
- ◯ Much worse
- ◯ Very much worse

2.01b Please check the option that best describes how your depressive disorder is now, compared with how it was before using medical cannabis.

- ◯ Very much better
- ◯ Much Better
- ◯ A little better
- ◯ No change
- ◯ A little worse
- ◯ Much worse
- ◯ Very much worse

2.01b Please check the option that best describes how your eating disorder is now, compared with how it was before using medical cannabis.

- ◯ Very much better
- ◯ Much Better
- ◯ A little better
- ◯ No change
- ◯ A little worse
- ◯ Much worse
- ◯ Very much worse

2.01b Please check the option that best describes how your manic disorder is now, compared with how it was before using medical cannabis.

- ◯ Very much better
- ◯ Much Better
- ◯ A little better
- ◯ No change
- ◯ A little worse
- ◯ Much worse
- ◯ Very much worse

2.01b Please check the option that best describes how your Post-Traumatic Stress disorder (PSTD) is now, compared with how it was before using medical cannabis.

- ◯ Very much better
- ◯ Much Better
- ◯ A little better
- ◯ No change
- ◯ A little worse
- ◯ Much worse
- ◯ Very much worse

2.01b Please check the option that best describes how your schizophrenia or other psychosis is now, compared with how it was before using medical cannabis.

- ◯ Very much better
- ◯ Much Better
- ◯ A little better
- ◯ No change
- ◯ A little worse
- ◯ Much worse
- ◯ Very much worse

2.01b Please check the option that best describes how your [q02_01a_condition_mentalh_other] is now, compared with how it was before using medical cannabis.

- ◯ Very much better
- ◯ Much Better
- ◯ A little better
- ◯ No change
- ◯ A little worse
- ◯ Much worse
- ◯ Very much worse

2.01a Please indicate which of the following neurological conditions you use medical cannabis to treat.

Please note: for the purposes of this survey fibromyalgia, neuropathy, and mental illnesses (e.g. depression, anxiety, ADHD, Schizophrenia) are NOT considered to be neurological disorders. If you clicked on this category in order to indicate one of these conditions you will find fibromyalgia and neuropathy under pain conditions and depression, anxiety, ADHD, and schizophrenia under mental health and/or substance use conditions.

- ☐ Amyotrophic Lateral Sclerosis
- ☐ Autism
- ☐ Dementia (including Alzheimer's)
- ☐ Epilepsy/ Seizure disorder
- ☐ Glaucoma
- ☐ Huntington's disease
- ☐ Multiple Sclerosis
- ☐ Parkinson's Disease
- ☐ Tourette's Syndrome
- ☐ Other neurological condition

(select all that apply (limit of three))

2.01a What other type of neurological conditions have you used medical cannabis to treat?

____
(please describe)

2.01b Have you been diagnosed with Amytrophic Lateral Sclerosis or had it confirmed in some way by a healthcare provider?

- ◯ Yes
- ◯ No

2.01b Have you been diagnosed with Autism or had it confirmed in some way by a healthcare provider?

- ◯ Yes
- ◯ No

2.01b Have you been diagnosed with Dementia or had it confirmed in some way by a healthcare provider?

- ◯ Yes
- ◯ No

2.01b Have you been diagnosed with Epilepsy or had it confirmed in some way by a healthcare provider?

- ◯ Yes
- ◯ No

2.01b Have you been diagnosed with Glaucoma or had it confirmed in some way by a healthcare provider?

- ◯ Yes
- ◯ No

2.01b Have you been diagnosed with Huntington's disease or had it confirmed in some way by a healthcare provider?

- ◯ Yes
- ◯ No

2.01b Have you been diagnosed with Multiple Sclerosis (MS) or had it confirmed in some way by a healthcare provider?

- ◯ Yes
- ◯ No

2.01b Have you been diagnosed with Parkinson's disease or had it confirmed in some way by a healthcare provider?

- ◯ Yes
- ◯ No

2.01b Have you been diagnosed with Tourette's syndrome or had it confirmed in some way by a healthcare provider?

- ◯ Yes
- ◯ No

2.01b Have you been diagnosed with [q02_01a_condition_neuro_other] or had it confirmed in some way by a healthcare provider?

- ◯ Yes
- ◯ No

2.01b Please check the option that best describes how your Amytrophic Lateral Sclerosis is now, compared with how it was before using medical cannabis.

- ◯ Very much better
- ◯ Much Better
- ◯ A little better
- ◯ No change
- ◯ A little worse
- ◯ Much worse
- ◯ Very much worse

2.01b Please check the option that best describes how your Autism is now, compared with how it was before using medical cannabis.

- ◯ Very much better
- ◯ Much Better
- ◯ A little better
- ◯ No change
- ◯ A little worse
- ◯ Much worse
- ◯ Very much worse

2.01b Please check the option that best describes how your Dementia is now, compared with how it was before using medical cannabis.

- ◯ Very much better
- ◯ Much Better
- ◯ A little better
- ◯ No change
- ◯ A little worse
- ◯ Much worse
- ◯ Very much worse

2.01b Please check the option that best describes how your Epilepsy is now, compared with how it was before using medical cannabis.

- ◯ Very much better
- ◯ Much Better
- ◯ A little better
- ◯ No change
- ◯ A little worse
- ◯ Much worse
- ◯ Very much worse

2.01b Please check the option that best describes how your Glaucoma is now, compared with how it was before using medical cannabis.

- ◯ Very much better
- ◯ Much Better
- ◯ A little better
- ◯ No change
- ◯ A little worse
- ◯ Much worse
- ◯ Very much worse

2.01b Please check the option that best describes how your Huntington's disease is now, compared with how it was before using medical cannabis.

- ◯ Very much better
- ◯ Much Better
- ◯ A little better
- ◯ No change
- ◯ A little worse
- ◯ Much worse
- ◯ Very much worse

2.01b Please check the option that best describes how your Multiple Sclerosis (MS) is now, compared with how it was before using medical cannabis.

- ◯ Very much better
- ◯ Much Better
- ◯ A little better
- ◯ No change
- ◯ A little worse
- ◯ Much worse
- ◯ Very much worse

2.01b Please check the option that best describes how your Parkinson's disease is now, compared with how it was before using medical cannabis.

- ◯ Very much better
- ◯ Much Better
- ◯ A little better
- ◯ No change
- ◯ A little worse
- ◯ Much worse
- ◯ Very much worse

2.01b Please check the option that best describes how your Tourette's syndrome is now, compared with how it was before using medical cannabis.

- ◯ Very much better
- ◯ Much Better
- ◯ A little better
- ◯ No change
- ◯ A little worse
- ◯ Much worse
- ◯ Very much worse

2.01b Please check the option that best describes how your [q02_01a_condition_neuro_other] is now, compared with how it was before using medical cannabis.

- ◯ Very much better
- ◯ Much Better
- ◯ A little better
- ◯ No change
- ◯ A little worse
- ◯ Much worse
- ◯ Very much worse

2.01a Please indicate which of the following pain conditions you have used medical cannabis to treat

- ☐ Arthritis (including rheumatoid or osteoarthritis)
- ☐ Back pain
- ☐ Cancer-related pain
- ☐ Fibromyalgia
- ☐ Headaches (including migraines)
- ☐ Menstrual pain
- ☐ Neck pain
- ☐ Neuropathy (nerve pain)
- ☐ Spinal cord injury
- ☐ Other type of chronic, non-cancer pain not described above

(select all that apply (limit of three))

2.01a What other type of pain disorder have you used medical cannabis to treat?

____
(please describe)

2.01b Have you been diagnosed with Arthritis or had it confirmed in some way by a healthcare provider?

- ◯ Yes
- ◯ No

2.01b Have you been diagnosed with back pain or had it confirmed in some way by a healthcare provider?

- ◯ Yes
- ◯ No

2.01b Have you been diagnosed with Cancer-related pain or had it confirmed in some way by a healthcare provider?

- ◯ Yes
- ◯ No

2.01b Have you been diagnosed with Fibromyalgia or had it confirmed in some way by a healthcare provider?

- ◯ Yes
- ◯ No

2.01b Have you been diagnosed with headaches or had it confirmed in some way by a healthcare provider?

- ◯ Yes
- ◯ No

2.01b Have you been diagnosed with menstrual pain or had it confirmed in some way by a healthcare provider?

- ◯ Yes
- ◯ No

2.01b Have you been diagnosed with neck pain or had it confirmed in some way by a healthcare provider?

- ◯ Yes
- ◯ No

2.01b Have you been diagnosed with neuropathy or had it confirmed in some way by a healthcare provider?

- ◯ Yes
- ◯ No

2.01b Have you been diagnosed with spinal cord injury or had it confirmed in some way by a healthcare provider?
- ◯ Yes
- ◯ No

2.01b Have you been diagnosed with this [q02_01a_condition_pain_other] or had it confirmed in some way by a healthcare provider?
- ◯ Yes
- ◯ No

2.01b Please check the option that best describes how your Arthritis is now, compared with how it was before using medical cannabis.
- ◯ Very much better
- ◯ Much Better
- ◯ A little better
- ◯ No change
- ◯ A little worse
- ◯ Much worse
- ◯ Very much worse

2.01b Please check the option that best describes how your back pain is now, compared with how it was before using medical cannabis.
- ◯ Very much better
- ◯ Much Better
- ◯ A little better
- ◯ No change
- ◯ A little worse
- ◯ Much worse
- ◯ Very much worse

2.01b Please check the option that best describes how your Cancer-related pain is now, compared with how it was before using medical cannabis.
- ◯ Very much better
- ◯ Much Better
- ◯ A little better
- ◯ No change
- ◯ A little worse
- ◯ Much worse
- ◯ Very much worse

2.01b Please check the option that best describes how your Fibromyalgia is now, compared with how it was before using medical cannabis.
- ◯ Very much better
- ◯ Much Better
- ◯ A little better
- ◯ No change
- ◯ A little worse
- ◯ Much worse
- ◯ Very much worse

2.01b Please check the option that best describes how your headaches are now, compared with how they were before using medical cannabis.
- ◯ Very much better
- ◯ Much Better
- ◯ A little better
- ◯ No change
- ◯ A little worse
- ◯ Much worse
- ◯ Very much worse

2.01b Please check the option that best describes how your menstrual pain is now, compared with how it was before using medical cannabis.
- ◯ Very much better
- ◯ Much Better
- ◯ A little better
- ◯ No change
- ◯ A little worse
- ◯ Much worse
- ◯ Very much worse

2.01b Please check the option that best describes how your neck pain is now, compared with how it was before using medical cannabis.

◯ Very much better
◯ Much Better
◯ A little better
◯ No change
◯ A little worse
◯ Much worse
◯ Very much worse

2.01b Please check the option that best describes how your neuropathy is now, compared with how it was before using medical cannabis.

◯ Very much better
◯ Much Better
◯ A little better
◯ No change
◯ A little worse
◯ Much worse
◯ Very much worse

2.01b Please check the option that best describes how pain from your spinal cord injury is now, compared with how it was before using medical cannabis.

◯ Very much better
◯ Much Better
◯ A little better
◯ No change
◯ A little worse
◯ Much worse
◯ Very much worse

2.01b Please check the option that best describes how your [q02_01a_condition_pain_other] is now, compared with how it was before using medical cannabis.

◯ Very much better
◯ Much Better
◯ A little better
◯ No change
◯ A little worse
◯ Much worse
◯ Very much worse

2.01a Please indicate which of the following sleep conditions you have used medical cannabis to treat

☐ Circadian rhythm sleep disorder (e.g. shift work type)
☐ Insomnia (any type)
☐ Narcolepsy or other hypersomnia
☐ Parasomnias (e.g. sleep walking, nightmares)
☐ Sleep apnoea or other sleep-related breathing disorder
☐ Sleep-related movement disorder (e.g. restless leg syndrome)
☐ Other sleep disorder
(select all that apply (limit of three))

2.01a What other type of sleep disorder do you use medical cannabis to treat?

____
(please describe)

2.01b Have you been diagnosed with circadian rhythm sleep disorder or had it confirmed in some way by a healthcare provider?

◯ Yes
◯ No

2.01b Have you been diagnosed with insomnia or had it confirmed in some way by a healthcare provider?

◯ Yes
◯ No

2.01b Have you been diagnosed with narcolepsy or had it confirmed in some way by a healthcare provider?

◯ Yes
◯ No

2.01b Have you been diagnosed with a parasomnia or had it confirmed in some way by a healthcare provider?

◯ Yes
◯ No

Have you been diagnosed with sleep-apnoea or had it confirmed in some way by a healthcare provider?
◯ Yes
◯ No

2.01b Have you been diagnosed with a sleep-related movement disorder or had it confirmed in some way by a healthcare provider?
◯ Yes
◯ No

2.01b Have you been diagnosed with [q02_01a_condition_sleep_other] or had it confirmed in some way by a healthcare provider?
◯ Yes
◯ No

2.01b Please check the option that best describes how your circadian rhythm sleep disorder is now, compared with how it was before using medical cannabis.
◯ Very much better
◯ Much Better
◯ A little better
◯ No change
◯ A little worse
◯ Much worse
◯ Very much worse

2.01b Please check the option that best describes how your insomnia is now, compared with how it was before using medical cannabis.
◯ Very much better
◯ Much Better
◯ A little better
◯ No change
◯ A little worse
◯ Much worse
◯ Very much worse

2.01b Please check the option that best describes how your narcolepsy is now, compared with how it was before using medical cannabis.
◯ Very much better
◯ Much Better
◯ A little better
◯ No change
◯ A little worse
◯ Much worse
◯ Very much worse

2.01b Please check the option that best describes how your Parasomnia is now, compared with how it was before using medical cannabis.
◯ Very much better
◯ Much Better
◯ A little better
◯ No change
◯ A little worse
◯ Much worse
◯ Very much worse

2.01b Please check the option that best describes how your sleep-apnoea is now, compared with how it was before using medical cannabis.
◯ Very much better
◯ Much Better
◯ A little better
◯ No change
◯ A little worse
◯ Much worse
◯ Very much worse

2.01b Please check the option that best describes how your sleep-related movement disorder is now, compared with how it was before using medical cannabis.
◯ Very much better
◯ Much Better
◯ A little better
◯ No change
◯ A little worse
◯ Much worse
◯ Very much worse

2.01b Please check the option that best describes how your [q02_01a_condition_sleep_other] is now, compared with how it was before using medical cannabis.

- ◯ Very much better
- ◯ Much Better
- ◯ A little better
- ◯ No change
- ◯ A little worse
- ◯ Much worse
- ◯ Very much worse

2.01a Please indicate which of the following other conditions you use medical cannabis to treat

- ☐ Auto-Immune condition (e.g. SLE, Chronic Fatigue disorder)
- ☐ Cardiovascular condition (e.g. poor circulation, ischemic heart disease)
- ☐ Diabetes Mellitus
- ☐ Gynaecological condition (e.g. endometriosis, PMS)
- ☐ HIV
- ☐ Infectious disease (e.g. viral hepatitis)
- ☐ Respiratory disease (e.g. asthma, cystic fibrosis)
- ☐ Skin condition (e.g. eczema, psoriasis, dermatitis)
- ☐ Other condition

(select all that apply (limit of five))

2.01a What other condition have you used medical cannabis to treat?

____
(please describe)

2.01b Have you been diagnosed with this Auto-immune condition or had it confirmed in some way by a healthcare provider?

- ◯ Yes
- ◯ No

2.01b Have you been diagnosed with this cardiovascular condition or had it confirmed in some way by a healthcare provider?

- ◯ Yes
- ◯ No

Have you been diagnosed with diabetes or had it confirmed in some way by a healthcare provider?

- ◯ Yes
- ◯ No

2.01b Have you been diagnosed with this gynaecological condition or had it confirmed in some way by a healthcare provider?

- ◯ Yes
- ◯ No

2.01b Have you been diagnosed with HIV or had it confirmed in some way by a healthcare provider?

- ◯ Yes
- ◯ No

2.01b Have you been diagnosed with this infectious disease or had it confirmed in some way by a healthcare provider?

- ◯ Yes
- ◯ No

2.01b Have you been diagnosed with this respiratory disease or had it confirmed in some way by a healthcare provider?

- ◯ Yes
- ◯ No

2.01b Have you been diagnosed with this skin condition or had it confirmed in some way by a healthcare provider?

- ◯ Yes
- ◯ No

2.01b Have you been diagnosed with [q02_01a_condition_other_other] or had it confirmed in some way by a healthcare provider?

- ◯ Yes
- ◯ No

2.01b Please check the option that best describes how your Auto-immune condition is now, compared with how it was before using medical cannabis.

- ◯ Very much better
- ◯ Much Better
- ◯ A little better
- ◯ No change
- ◯ A little worse
- ◯ Much worse
- ◯ Very much worse

2.01b Please check the option that best describes how your cardiovascular condition is now, compared with how it was before using medical cannabis.

- ◯ Very much better
- ◯ Much Better
- ◯ A little better
- ◯ No change
- ◯ A little worse
- ◯ Much worse
- ◯ Very much worse

2.01b Please check the option that best describes how your diabetes is now, compared with how it was before using medical cannabis.

- ◯ Very much better
- ◯ Much Better
- ◯ A little better
- ◯ No change
- ◯ A little worse
- ◯ Much worse
- ◯ Very much worse

2.01b Please check the option that best describes how your gynaecological condition is now, compared with how it was before using medical cannabis.

- ◯ Very much better
- ◯ Much Better
- ◯ A little better
- ◯ No change
- ◯ A little worse
- ◯ Much worse
- ◯ Very much worse

2.01b Please check the option that best describes how your HIV is now, compared with how it was before using medical cannabis.

- ◯ Very much better
- ◯ Much Better
- ◯ A little better
- ◯ No change
- ◯ A little worse
- ◯ Much worse
- ◯ Very much worse

2.01b Please check the option that best describes how your infectious disease is now, compared with how it was before using medical cannabis.

- ◯ Very much better
- ◯ Much Better
- ◯ A little better
- ◯ No change
- ◯ A little worse
- ◯ Much worse
- ◯ Very much worse

2.01b Please check the option that best describes how your respiratory disease is now, compared with how it was before using medical cannabis.

- ◯ Very much better
- ◯ Much Better
- ◯ A little better
- ◯ No change
- ◯ A little worse
- ◯ Much worse
- ◯ Very much worse

2.01b Please check the option that best describes how your skin condition is now, compared with how it was before using medical cannabis.

- ◯ Very much better
- ◯ Much Better
- ◯ A little better
- ◯ No change
- ◯ A little worse
- ◯ Much worse
- ◯ Very much worse

2.01b Please check the option that best describes how your [q02_01a_condition_other_other] is now, compared with how it was before using medical cannabis.

- ◯ Very much better
- ◯ Much Better
- ◯ A little better
- ◯ No change
- ◯ A little worse
- ◯ Much worse
- ◯ Very much worse

2.01c The following questions relate to your sleep habits WHILE USING MEDICAL CANNABIS DURING THE PAST MONTH ONLY.

Your answers should indicate the most accurate reply for the majority of days and nights in the past month.

During the past month...

2.01c When have you usually gone to bed at night?

Please answer in 24-HOUR TIME
E.g., 9pm = 21:00; 10pm = 22:00, 10.30pm = 22:30, 11pm = 23:00, 12am = 00:00, 1.15am = 01:15

____
(Answer in 24-h time, e.g. 15:30)

2.01c How long has it usually taken you to fall asleep each night?

Please answer in MINUTES

____
(Answer in minutes)

2.01c How many hours of actual sleep did you get at night?

Please answer in HOURS.

____
(Answer in hours)

2.01c Have you had trouble sleeping because you wake up in the middle of the night or early morning?

- ◯ Not during the past month
- ◯ Less than once a week
- ◯ Once or twice a week
- ◯ Three or more time a week

2.01c How would you rate your sleep quality overall?

- ◯ Very good
- ◯ Fairly good
- ◯ Fairly bad
- ◯ Very bad

Of the conditions you listed above, which is the main condition you treat with medical cannabis. If you only indicated one health condition above, please just select it again below. The main health condition I use medical cannabis for is:

	MAIN condition
Addiction (Cannabis)	○
Addiction (Other drugs: including alcohol, opioid, amphetamine)	○
Amyotrophic Lateral Sclerosis	○
Anxiety Disorder (e.g. generalised anxiety, panic disorder, OCD)	○
Arthritis (including rheumatoid or osteoarthritis	○
Attention Deficit disorder (ADHD)	○
Autism	○
Auto-Immune condition (e.g. SLE, chronic fatigue disorder)	○
Back pain	○
Blood cancers (leukaemia, lymphoma)	○
Brain cancers (glioblastoma, neuroblastoma, mesothelioma)	○
Breast cancer	○
Cancer-related pain	○
Cardiovascular condition (e.g. poor circulation, ischemic heart disease)	○
Circadian rhythm sleep disorder (e.g. shift work type)	○
Crohn's disease	○
Dementia (including Alzheimer's)	○
Depression	○
Diabetes Mellitus	○
Eating disorders (e.g. anorexia / bulimia/ obesity)	○
Epilepsy/ Seizure disorder	○
Fibromyalgia	○
Gastrointestinal cancer (bowel, colon, stomach)	○
Glaucoma	○

Gynaecological condition (e.g. endometriosis) ○

Headaches (including migraines) ○

HIV ○

Huntington's disease ○

Infectious disease (e.g. viral hepatitis) ○

Insomnia (any type) ○

Irritable bowel syndrome ○

Lung cancer ○

Manic Disorder (Bipolar affective disorder) ○

Menstrual pain ○

Multiple Sclerosis ○

Narcolepsy or other hypersomnia ○

Neck pain ○

Neuropathy (nerve pain) ○

[q02_01a_condition_cancer_other] ○

[q02_01a_condition_gastro_other] ○

[q02_01a_condition_mentalh_other] ○

[q02_01a_condition_neuro_other] ○

[q02_01a_condition_sleep_other] ○

[q02_01a_condition_pain_other] ○

Parasomnias (e.g. sleep walking, nightmares) ○

Parkinson's disease ○

Post-Traumatic Stress Disorder ○

Reproductive cancers (cervical, uterine, testicular, ovarian) ○

Respiratory disease (e.g. asthma, cystic fibrosis) ○

Schizophrenia or other psychosis ○

Skin cancers (melanoma) ○

Skin condition (e.g. eczema, psoriasis, dermatitis) ○

Sleep apnoea or other sleep-related breathing disorder ○

Sleep-related movement disorder (e.g. restless leg syndrome) ○

Spinal cord injury ○

Tourette's Syndrome	◯
Ulcerative colitis	◯
[q02_01a_condition_other_other]	◯

Survey Progress
34%

SECTION 3

CANNABIS AND OTHER DRUG USE

Please provide us with some information about how you use cannabis and other drugs

3.01 How old were you when you first tried cannabis for MEDICAL purposes?

____
(years old (in numbers))

3.02 How old were you when you first started using cannabis REGULARLY for MEDICAL purposes? (at least once per week for a period of three months or longer).

If you have never used cannabis REGULARLY for MEDICAL reasons please enter '0'

____
(years old (in numbers). If you have never used cannabis regularly for medical reasons please enter '0')

____

____

You said you FIRST TRIED cannabis for MEDICAL purposes when you were [q03_01_age_tried_medical] years-old but that you first started using cannabis REGULARLY for MEDICAL purposes when you were [q03_02_regular_use_medical] years-old. This does not make sense. You can't do something regularly before you do it for the first time. The age when you first tried medical cannabis must be the same age or younger than the age you first started using medical cannabis regularly. Please think carefully and adjust your answers accordingly.

3.03 In the last 28 days, please estimate how many days you used cannabis for MEDICAL reasons:

If you have not used cannabis for medical reasons in the last 28 days, please enter '0'

____
(enter (0-28))

You have indicated you have used cannabis for medical purposes 0 days in the last 28. Please answer the following questions in relation to your medical cannabis use during the last period when you were using medical cannabis.

3.03a On an average day that you use cannabis for MEDICAL reasons, on how many occasions do you usually use cannabis for MEDICAL reasons (e.g. number of times per day)?

____
((max 20))

3.04 Please estimate the cost of your medical cannabis on an average WEEKLY basis in Australian dollars.

If you do not purchase your medical cannabis please enter zero (0).

____
(amount per week in dollars)

3.05 Have you ever used cannabis for non-medical (i.e. recreational) reasons?

◯ Yes
◯ No

3.06 Which of the following best describes your use of cannabis before you used it for medical reasons?

◯ I had not used cannabis non-medically before I started using it medically
◯ I had used cannabis for non-medical reasons but had quit for a year or more before taking up cannabis use for medical reasons.
◯ I was using cannabis for non-medical purposes at the time when I started using it medically
(select one option)

3.06a Please estimate the time between your last non-medical use of cannabis and starting medical cannabis

Please enter answer in number of MONTHS?

1 year = 12
1.5 years = 18
2 years = 24
5 years = 60
10 years = 120
20 years = 240

____
(number of months)

____

You said that you had quit non-medical cannabis use for a year or more before you you took up medical use, but in the last question you said the time between non-medical and medical use was [q03_06a_time_between_rec_and_med_use] months.

If the gap between your non-medical and medical use was LESS than a year please go back and answer 'I was using cannabis for non-medical purposes at the time when I started using it medically' to question 3.06.

If the gap was MORE than a year, please enter a value of 12 or more for the previous question (3.06a)

3.06b Have you started using cannabis non-medically (i.e. recreationally) again since you started using it medically?

◯ Yes
◯ No

3.06c How many MONTHS after you started medical cannabis use did you resume non-medical (i.e. recreational) use?

2 weeks = 0.5
0.5 years = 6
1 year = 12
1.5 years = 18
2 years = 24
5 years = 60

____
(Please answer in months)

3.07 How old were you when you first tried cannabis for NON-MEDICAL (e.g. recreational) reasons?

____
(years old (in numbers))

3.08 How old were you when you first started using cannabis REGULARLY (at least once per week for a period of three months or longer) for NON-MEDICAL (e.g. recreational) reasons?

If you have never used cannabis REGULARLY for NON-MEDICAL reasons please enter '0'

____
(years old (in numbers). If you have never used cannabis regularly please enter '0')

____

____

You have said that you were [q03_07_age_tried_nonmed] years-old when you FIRST TRIED cannabis for NON-MEDICAL REASONS but that you were [q03_08_regular_use_nonmed] years-old when you started using cannabis REGULARLY for NON-MEDICAL reasons. This does not make sense. You can't do something regularly before you do it for the first time. The age you first tried cannabis for non-medical reasons must be the same age or younger than the age you started using cannabis regularly for non-medical reasons. Please think carefully and adjust your answers accordingly.

____

You have indicated that you did not use cannabis NON-MEDICALLY before you began using it MEDICALLY, yet the age you said that you started using cannabis REGULARLY for NON-MEDICAL reasons ([q03_08_regular_use_nonmed] years-old) is younger than the age you said you started using REGULARLY for MEDICAL reasons ([q03_02_regular_use_medical] years-old). This does not make sense. Please think about your answers to these questions carefully and adjust your previous answers accordingly.

3.09 In the last 28 days, please estimate how many days you used cannabis for NON-MEDICAL (e.g. recreational) reasons.

If you have not used cannabis for non-medical reasons in the past 28 days, please enter '0'

____
(enter (0-28))

You have indicated you have used cannabis for non-medical purposes 0 days in the last 28. Please answer the following questions in relation to your non-medical cannabis use during the last period when you were using cannabis for non-medical reasons.

____

3.09a On an average day that you use cannabis for NON-MEDICAL reasons, on how many occasions do you usually use cannabis for NON-MEDICAL reasons (e.g. number of times per day)?

____
((max 20))

____

3.10 On how many of the last 28 days have you used cannabis for ANY reason (i.e. either MEDICAL or NON-MEDICAL)?

If you have not used cannabis for ANY reason in the last 28 days please enter '0'

____
(enter (0-28))

____

You have said that you used cannabis for MEDICAL reasons [q03_03_usage_days_medical] days in the last 28 but that you used cannabis for ANY reason [q03_10_usage_days_any] days. This does not make sense. Days' use for MEDICAL reasons can only ever be equal to or less than days' use for ANY reason. Please adjust your answers to these questions accordingly.

____

You have said that you used cannabis for NON-MEDICAL reasons [q03_09_usage_days_nonmed] days in the last 28 but that you used cannabis for ANY reason [q03_10_usage_days_any] days. This does not make sense. Days' use for NON-MEDICAL reasons can only ever be equal to or less than days' use for ANY reason. Please adjust your answers to these questions accordingly.

____

The number of days you said you used cannabis for ANY reason ([q03_10_usage_days_any] days) is greater than the combined value you entered for number of days using cannabis for MEDICAL reasons PLUS number of days using for NON-MEDICAL reasons ([q03_03_usage_days_medical] + [q03_09_usage_days_nonmed] = [daysmedplusnonmed] days). The maximum number of days' use for ANY reason can only be the same or less than the combined number of days for MEDICAL and NON-MEDICAL reasons. Please reconsider your answers to these questions and adjust your responses accordingly.

3.10a On an average day that you use cannabis for ANY reason, on how many occasions do you usually use cannabis (e.g. number of times per day)?

____
((max 20))

____

You have said that you use cannabis for MEDICAL reasons [q03_03a_times_use_per_day_medical] times per day on average, but that you used cannabis [q03_10a_times_use_per_day_any] times per day for ANY reason (i.e. including medical). This does not make sense. Number of occasions of MEDICAL use per day can only ever be equal to or less than number of occasions per day for ANY reason. Please adjust your answers to these questions accordingly.

____

You have said that you use cannabis for NON-MEDICAL reasons [q03_09a_times_use_per_day_nonmed] times per day on average, but that you used cannabis [q03_10a_times_use_per_day_any] times per day for ANY reason (i.e. including non-medical). This does not make sense. Number of occasions of NON-MEDICAL use per day can only ever be equal to or less than number of occasions per day for ANY reason. Please adjust your answers to these questions accordingly.

____

Your entry for the number of times per day you use cannabis for ANY reason ([q03_10a_times_use_per_day_any] times per day) is greater than the combined value you entered for number of times per day you use cannabis for MEDICAL reasons PLUS the number times per day you use for NON-MEDICAL reasons ([q03_03a_times_use_per_day_medical] + [q03_09a_times_use_per_day_nonmed] = [occasionsmedplusnonmed] times per day). This does not make sense. Number of times per day used for ANY reason can only be equal to or less than the combined times per day for MEDICAL and NON-MEDICAL reasons. Please reconsider your answers to these questions and adjust your responses accordingly.

3.11 Please indicate what percentage of your TOTAL cannabis use in the last 12 months use you consider to be MEDICAL use.

In other words what percentage of your TOTAL use (MEDICAL plus NON-MEDICAL) is MEDICAL?

0% 50% 100%

(Place a mark on the scale above)

____

You have indicated that you the percentage of your cannabis use that is for medical purposes is 0%. If this is true you are ineligible for the study. Please reconsider your answer to this question and adjust accordingly.

3.12 Please estimate the TOTAL cost of your cannabis (i.e. medical PLUS non-medical) on an average WEEKLY basis in Australian dollars.

If you do not purchase your cannabis please enter zero (0).

____
(amount per week in dollars)

____

You have indicated that the weekly cost of your MEDICAL cannabis use is $[q03_04_cost_in_dollars_med] but that the TOTAL weekly cost is $[q03_12_cost_in_dollars_total]. This does not make sense. The cost of your medical use can only be LESS THAN or EQUAL to the cost of your total use. Please consider your answers carefully and answer again.

____

You have indicated that [q03_11_percentage_medical]% of your cannabis use is for medical purposes but that you have used cannabis for NON-MEDICAL purposes [q03_09_usage_days_nonmed] days in the last 28. This does not make sense. Please consider your answers carefully and answer again.

____

You have indicated that [q03_11_percentage_medical]% of your cannabis use is for medical purposes but that the TOTAL cost of your cannabis is greater than the MEDICAL cost. This does not make sense. If you used no cannabis for NON-MEDICAL reasons then the TOTAL weekly cost of your cannabis and the MEDICAL COST should be the same. Please consider your answers carefully and answer again.

3.13 Did your cannabis use change as a result of the COVID-19 pandemic?

- ◯ Yes, my cannabis use increased
- ◯ No, my cannabis use did not change
- ◯ Yes, my cannabis use decreased

(Select one option)

3.14 On how many of the last 28 days have you consumed tobacco?

____
((0-28))

3.14a Please estimate how many cigarettes (of tobacco) you currently consume per day on days when you consume tobacco.

____
(number of cigarettes per day)

3.14b Since starting medical cannabis my use of tobacco has:

- ◯ Markedly decreased (by 50% or more)
- ◯ Moderately decreased (by 20-50%)
- ◯ More or less stayed the same (no change greater than 20% each way)
- ◯ Moderately increased (by 20-50%)
- ◯ Markedly increased (by 50% or more)

(select one option)

3.14c Do you usually mix tobacco with your medical cannabis?

- ◯ Yes, I always mix tobacco with my medical cannabis
- ◯ Yes, I sometimes mix tobacco with my medical cannabis
- ◯ No, I never mix tobacco with my medical cannabis

3.15 On how many of the last 28 days did you consume alcohol?

____
((0-28))

3.15a Please estimate how many standard drinks you consumed per day on average (on days when you drank).

Guide:
285 ml of beer, 425 of light beer, 100ml of wine, 60ml of fortified wine and 30 ml of spirits, all = 1 standard drink.

A full-strength can or stubby of beer = 1.5 standard drinks.

____
(In standard drinks.)

3.15b Since starting use of medical cannabis, my use of alcohol has:

- ◯ Markedly decreased (by 50% or more)
- ◯ Moderately decreased (by 20-50%)
- ◯ More or less stayed the same (no change greater than 20% each way)
- ◯ Moderately increased (by 20-50%)
- ◯ Markedly increased (by 50% or more)

(select one option)

Survey Progress
43%

SECTION 4

ACCESSING MEDICAL CANNABIS

In the previous section (Section 3) we asked you about your use of cannabis, both for MEDICAL reasons (to treat a health condition) and for NON-MEDICAL reasons (e.g. 'recreational' use).

In the next section we will ask you about how you access your MEDICAL cannabis, the cannabis you use for MEDICAL reasons. We are especially interested whether you get your MEDICAL cannabis via LEGAL routes (by getting a prescription) or through ILLICIT routes (e.g. from a dealer, friend, or family member).

It is important to remind you that your responses are anonymous and confidential.

4.00 Why did you start using medical cannabis? Please select all options that apply.

- ☐ My usual medications were not working on my long-term or chronic illness
- ☐ I preferred a natural alternative to my usual medication
- ☐ Side effects of my usual medications
- ☐ While using recreationally I discovered cannabis had medicinal benefits
- ☐ It was recommended to me by a friend or family member
- ☐ It was recommended to me by a health professional (GP, specialist, allied health, holistic etc)
- ☐ I had a willingness to explore alternative health management strategies
- ☐ Other

4.00a Please tell us the other reason you started using medical cannabis

____

4.01 Have you ever accessed legal (prescribed) medical cannabis prescribed by a doctor in Australia?

Prescribed medical cannabis products are obtained by filling a script at a pharmacy or a chemist.

- ◯ Yes
- ◯ No

4.01 How long ago was it that you FIRST started using legal (prescribed) medical cannabis?

Please answer in YEARS

If you first started using legal (prescribed) medical cannabis LESS than a year ago please enter what fraction of a year. For example, if you started legal (prescribed) medical cannabis six months ago enter '0.5', if 3 months ago '0.25', if 1 month ago '0.08', if two weeks '0.04' etc.

____
(answer in years)

____

You have indicated that you were first prescribed medical cannabis [q04_01a_whenfirstprescribed] years ago. Medical cannabis was first legalised in October 2016, which means you are saying you were prescribed legal medical cannabis before it was legal. This does not make sense. Please reconsider your answers and respond accordingly.

4.01 In the last 28 days on how many days did you use legal (prescribed) medical cannabis?

If you did not use PRESCRIBED medical cannabis in the last 28 days please enter '0'

____
(answer in days)

____

4.01a Who is the main prescriber of your medical cannabis?

- ◯ General practitioner (GP) in a general health setting
- ◯ Medical specialist in a general health setting (e.g. neurologist, pain specialist)
- ◯ General practitioner (GP) in a cannabis clinic
- ◯ Medical specialist in a cannabis clinic

4.01a What is the name of the MAIN private clinic you use to access your prescribed medical cannabis?

- ◯ Cannabis Access Clinics (CA Clinics)
- ◯ Medical Cannabis Services (MCS Clinics)
- ◯ Cannabis Doctors Australia (CDA Clinics)
- ◯ Tetra Health Clinics
- ◯ Other

4.01a What is the name of the other private medical cannabis clinic you use to access your medical cannabis?

____

4.01a What type(s) of medical cannabis are you prescribed?

- ☐ THC only
- ☐ Mainly THC and small amounts of other cannabinoids (e.g. CBD)
- ☐ Approximately equal amounts of THC and CBD
- ☐ Mainly CBD and small amounts of other cannabinoids (e.g. THC)
- ☐ CBD only
- ☐ Unsure/Do not know
- ☐ It varies between batches

(Select all that apply)

4.01a Of the types of medical cannabis you selected in the previous question, which is the main type you use?

Note: If you only selected one option above, please select it again below.

	Select one option
THC Only	◯
Mainly THC and small amounts of other cannabinoids (e.g. CBD)	◯
Approximately equal amounts of THC and CBD	◯
Mainly CBD and small amounts of other cannabinoids (e.g. THC)	◯
CBD only	◯

4.01a What ways do you consume your prescribed medical cannabis?

- ☐ Oral (tablet or capsule)
- ☐ Oral edibles (cake, cookie etc.)
- ☐ Oral liquid concentrate (oil, tincture, etc.)
- ☐ Oral mouth spray
- ☐ Nasal application (through nose)
- ☐ Smoked as a 'joint' (cigarette)
- ☐ Smoked using a 'dry' pipe (plastic, metal, glass)
- ☐ Smoked using a water pipe or 'Bong'
- ☐ Smoked by 'dabbing' or 'spotting'
- ☐ Inhaled herb using a vaporiser
- ☐ Inhaled liquid using a vaporiser
- ☐ Other

(Select all that apply)

4.01a What other ways have you consumed prescribed medical cannabis?

____

4.01a Of the ways of consuming legal (prescribed) medical cannabis you listed above, which is the main way?

Note: If you only listed one option in the last question, please select it again below

	Select one option
Oral (tablet or capsule)	◯
Oral edibles (cake, cookie etc)	◯
Oral liquid concentrate (oil, tincture etc)	◯
Oral mouth spray	◯
Nasal application	◯
Smoked as a 'joint' (cigarette)	◯
Smoked using a dry pipe (plastic, metal, glass)	◯
Smoked using a water pipe or bong	◯
Smoked by dabbing or spotting	◯
Inhaled herb using a vaporiser	◯
Inhaled liquid using a vaporiser	◯
[q04_01a_preferred_form_other]	◯

4.01a Do you feel that the quality or composition of your prescribed medical cannabis is consistent over time?

◯ Yes
◯ No
◯ Unsure/Don't know
(Select one option)

4.01a Since you started using legal (prescribed) medical cannabis have you ever been unable to access your preferred source of legal (prescribed) medical cannabis to treat your health concerns?

◯ Yes, frequently
◯ Yes, occasionally
◯ No

4.01a Are you concerned about the possibility of contaminants in your legal (prescribed) medical cannabis (e.g. heavy metals, toxins, pesticides, bacteria/fungi/mould)?

◯ A great deal
◯ Quite a bit
◯ A little
◯ Not at all
◯ I'm not sure
(select one option)

4.01a How much does your legal (prescribed) medical cannabis medication cost on an average week?

Please estimate the cost of your medication only, not any other costs associated with obtaining it (e.g. doctors' fees, delivery costs)

____
(answer in whole dollars (e.g. 20))

4.01a Please estimate the average amount, in dollars, you would spend on other costs associated with obtaining your medical cannabis in an average week.

By other costs we mean doctors' fees, delivery costs etc (i.e. all costs other than medication)

____
(answer in whole dollars (e.g. 20))

4.01 Have you ever been reimbursed for the cost of your prescribed medical cannabis medication (e.g. by the Department for Veterans affairs, Workers Compensation, or by a private health insurer)?

◯ No, I have never been reimbursed for my medical cannabis expenses
◯ Yes, by the Department of Veterans Affairs
◯ Yes, by Workers compensation
◯ Yes, by Private Health Insurance
◯ Yes by another source
(Select one option)

4.01a Which other agency reimbursed you for the cost of your prescribed medical cannabis medication?

____

4.01a On the occasions where you were reimbursed for your prescribed medical cannabis medication, what percentage of the cost did you get back?

0% 50% 100%

(Place a mark on the scale above)

4.01a Please indicate your level of satisfaction with the treatment you received from your medical cannabis prescriber

	Very satisfied	Satisfied	Neutral	Dissatisfied	Very dissatisfied
The amount of information provided to me by my doctor about the possible benefits and side effects of medical cannabis	○	○	○	○	○
The amount of information provided to me by my doctor about the evidence for using medical cannabis to treat my health condition	○	○	○	○	○
The amount of time the doctor took to do the initial assessment	○	○	○	○	○
The amount of time the doctor takes in follow up appointments	○	○	○	○	○
My doctor addresses any questions or concerns I have about medical cannabis	○	○	○	○	○
My doctor includes other approaches to treat the condition I use medical cannabis for (e.g. other medications, referral to counselling, physiotherapy, surgery etc...)	○	○	○	○	○
The fees my doctor charges	○	○	○	○	○

4.01a Does the doctor who prescribes your medical cannabis also treat your other health problems (unrelated to medical cannabis)

Please tick the option that best describes your situation

- ○ Yes - my medical cannabis doctor treats all my health issues (including health issues I do not use medical cannabis for)
- ○ No - I go to other doctors for other health issues
- ○ I don't have any other health problems

(Select one option)

____

You indicated above that you have used legal (prescribed) medical cannabis in the past, but that you have not accessed legal (prescribed) medical cannabis in the last 28 days.

The following questions will be about your use of prescribed medical cannabis during the period when you were being prescribed and using it.

4.01b Who was the main prescriber of your medical cannabis?

◯ General practitioner (GP) in a general health setting
◯ Medical specialist in a general health setting (e.g. neurologist, pain specialist)
◯ General practitioner (GP) in a cannabis clinic
◯ Medical specialist in a cannabis clinic
(select one option)

4.01b What was the name of the MAIN private clinic you used to access your prescribed medical cannabis?

◯ Cannabis Access Clinics (CA Clinics)
◯ Medical Cannabis Services (MCS Clinics)
◯ Cannabis Doctors Australia (CDA Clinics)
◯ Tetra Health Clinics
◯ Other

4.01b What was the name of the other private medical cannabis clinic you use to access your medical cannabis?

____

4.01b How long ago did you STOP using legal (prescribed) medical cannabis?

Please enter answer in number of MONTHS?

6 weeks = 1.5
3 months = 3
1 year = 12
2 years = 24
3 years = 36

____
(in months (please use numbers).)

____

You have indicated that you STOPPED prescribed medical cannabis [q04_01b_howlongagostoppedprescribed] months ago. Medical cannabis was first legalised in October 2016, which means you are saying you STOPPED legal medical cannabis before it was legal. This does not make sense. Please reconsider your answers and respond accordingly.

____

In the previous question you indicated that you stopped accessing legal (prescribed) medical cannabis less than one month ago, but in a previous question you answered that you used 0 days of legal (prescribed) cannabis in the last 28 days. If you DID use legal (prescribed) cannabis in the last 28 days please go back to that question and enter how many days you used. If you did NOT use legal (prescribed) medical cannabis in the last 28 days please give an answer greater than '1' for the previous question?'

4.01b Please tell us the reasons you stopped using legal (prescribed) medical cannabis.

- ☐ Prescribed medical cannabis wasn't improving my symptoms
- ☐ Prescribed medical cannabis supplies became too expensive for me to afford
- ☐ Prescribed medical cannabis became difficult to access
- ☐ I became concerned about mobile drug-testing
- ☐ I became concerned about side-effects
- ☐ My medical cannabis practitioner became unwilling to keep prescribing
- ☐ I preferred using illicit cannabis
- ☐ I preferred to keep my medical cannabis use confidential from healthcare providers
- ☐ Prescribed medical cannabis was too expensive for me to continue
- ☐ Other

(Select all that apply)

4.01b What other reason made you stop using legal (prescribed) medical cannabis?

____

4.01b For how long had you been accessing legal (prescribed) medical cannabis before you stopped?

Please enter answer in number of MONTHS?

6 weeks = 1.5
3 months = 3
1 year = 12
2 years = 24
3 years = 36

____
(in months (please use numbers). If less than one month please indicate, e.g. 2 weeks = 0.5 months, 3 weeks = 0.75 months)

____

You have indicated that you had been using legal (prescribed) medical cannabis for [q04_01b_durationprescribedbeforestopped] months before you stopped. Medical cannabis was first legalised in October 2016, which means you are saying you had been using legal medical cannabis for more time than it has been legal BEFORE you STOPPED. This does not make sense. Please reconsider your answers and respond accordingly.

4.01b What type(s) of medical cannabis were you prescribed?

- ☐ THC only
- ☐ Mainly THC and small amounts of other cannabinoids (e.g. CBD)
- ☐ Approximately equal amounts of THC and CBD
- ☐ Mainly CBD and small amounts of other cannabinoids (e.g. THC)
- ☐ CBD only
- ☐ Unsure/Do not know
- ☐ It varies between batches

(Select all that apply)

4.01b Of the types of medical cannabis you selected in the previous question, what was the main type you used?

Note: If you only selected one option above, please select it again below.

	Select one option
THC Only	◯
Mainly THC and small amounts of other cannabinoids (e.g. CBD)	◯
Approximately equal amounts of THC and CBD	◯
Mainly CBD and small amounts of other cannabinoids (e.g. THC)	◯
CBD only	◯

4.01b Please list the ways you consumed your legal (prescribed) medical cannabis?

- ☐ Oral (tablet or capsule)
- ☐ Oral edibles (cake, cookie etc.)
- ☐ Oral liquid concentrate (oil, tincture, etc.)
- ☐ Oral mouth spray
- ☐ Nasal application (through nose)
- ☐ Smoked as a 'joint' (cigarette)
- ☐ Smoked using a 'dry' pipe (plastic, metal, glass)
- ☐ Smoked using a water pipe or 'Bong'
- ☐ Smoked by 'dabbing' or 'spotting'
- ☐ Inhaled herb using a vaporiser
- ☐ Inhaled liquid using a vaporiser
- ☐ Other

(Select all that apply)

4.01b What other way have you consumed legal (prescribed) medical cannabis?

____

4.01b Of the ways of consuming legal (prescribed) medical cannabis you listed above, which was the main way?

Note: If you only listed one option in the last question, please select it again below

	Select one option
Oral (tablet or capsule)	◯
Oral edibles (cake, cookie etc)	◯
Oral liquid concentrate (oil, tincture etc)	◯
Oral mouth spray	◯
Nasal application	◯
Smoked as a 'joint' (cigarette)	◯
Smoked using a dry pipe (plastic, metal, glass)	◯
Smoked using a water pipe or bong	◯
Smoked by dabbing or spotting	◯
Inhaled herb using a vaporiser	◯
Inhaled liquid using a vaporiser	◯
[q04_01b_preferred_form_other]	◯

4.01b When you were accessing legal (prescribed) medical cannabis did you feel that the quality or composition of your legal (prescribed) medical cannabis was consistent over time?

- ◯ Yes
- ◯ No
- ◯ Unsure/Don't know

(Select one option)

4.01b When you were accessing legal (prescribed) medical cannabis were you ever unable to access your preferred source of prescribed medical cannabis to treat your health concerns?

◯ Yes, frequently
◯ Yes, occasionally
◯ No

4.01b When you were accessing legal (prescribed) medical cannabis, were you ever concerned about the possibility of contaminants in your prescribed medical cannabis (e.g. heavy metals, toxins, pesticides, bacteria/fungi/mould)?

◯ A great deal
◯ Quite a bit
◯ A little
◯ Not at all
◯ I'm not sure
(select one option)

4.01b When you were accessing legal (prescribed) medical cannabis how much did your medication cost on average each week?

Please estimate the cost of your medication only, not any other costs associated with obtaining it (e.g. consultation fees with prescribers, delivery costs)

____
(answer in whole dollars (e.g. 20))

4.01b When you were accessing legal (prescribed) medical cannabis, please estimate the average amount, in dollars, you would spend on other costs associated with obtaining your medical cannabis in an average week.

By other costs we mean consultation fees, delivery costs etc (i.e. all costs other than medication)

____
(answer in whole dollars (e.g. 20))

4.01b Have you ever been reimbursed for the cost of your prescribed medical cannabis medication (e.g. by the Department for Veterans affairs, Workers Compensation, or by a private health insurer)?

◯ No, I have never been reimbursed for my medical cannabis expenses
◯ Yes, by the Department of Veterans Affairs
◯ Yes, by Workers compensation
◯ Yes, by Private Health Insurance
◯ Yes by another source
(Select all that apply)

4.01b Which other agency reimbursed you for the cost of your prescribed medical cannabis medication?

____

4.01b On the occasions where you were reimbursed for your prescribed medical cannabis medication, what percentage of the that cost did you get back?

0% 50% 100%

(Place a mark on the scale above)

4.01b Please indicate your level of satisfaction with the treatment you received from your medical cannabis prescriber (when you were receiving it)

	Very satisfied	Satisfied	Neutral	Dissatisfied	Very dissatisfied
The amount of information provided to me by my doctor about the possible benefits and side effects of medical cannabis	◯	◯	◯	◯	◯

The amount of information provided to me by my doctor about the evidence for using medical cannabis to treat my health condition	○	○	○	○	○
The amount of time the doctor took to do the initial assessment	○	○	○	○	○
The amount of time the doctor took in follow up appointments	○	○	○	○	○
My doctor addressed any questions or concerns I had about medical cannabis	○	○	○	○	○
My doctor included other approaches to treat the health conditions I used medical cannabis for (e.g. other medications, referral to counselling, physiotherapy, surgery etc...)	○	○	○	○	○
The fees my doctor charged	○	○	○	○	○

4.01b You have indicated that you have never accessed legal medical cannabis prescribed by a doctor in Australia.

Please describe the main reasons for you not accessing medical cannabis from a medical practitioner

- ☐ I did not know I could legally access medical cannabis from a doctor
- ☐ I do not know a medical practitioner who is willing to prescribe medical cannabis
- ☐ My medical practitioner is not interested or is unwilling to prescribe
- ☐ Medical cannabis supplies are too expensive for me to afford
- ☐ I prefer using illicit cannabis
- ☐ I prefer to keep my medical cannabis use confidential from healthcare providers
- ☐ Other reason

(You can select more than one option.)

4.01b Please describe the other reason you have never accessed legal (prescribed) medical cannabis

____

4.01b If you did choose to access prescribed medical cannabis, what would be the MAIN way you would prefer to consume it?

- ○ Oral (tablet or capsule)
- ○ Oral edibles (cake, cookie etc.)
- ○ Oral liquid concentrate (oil, tincture, etc.)
- ○ Oral mouth spray
- ○ Nasal application (through nose)
- ○ Smoked as a 'joint' (cigarette)
- ○ Smoked using a 'dry' pipe (plastic, metal, glass)
- ○ Smoked using a water pipe or 'Bong'
- ○ Smoked by 'dabbing' or 'spotting'
- ○ Inhaled using a vaporiser
- ○ Other

(select one option)

What other way would you prefer to consume your prescribed medical cannabis?

____

4.01b Please estimate how much you would be prepared to spend each week on your prescribed medical cannabis?

Please answer in whole dollars.

____
(in whole dollars)

4.01a Did the doctor who prescribed your medical cannabis also treat your other health problems (unrelated to medical cannabis)?

Please tick the option that best describes your situation

- ◯ Yes - my medical cannabis doctor treated all my health issues (including health issues I did not use medical cannabis for)
- ◯ No - I went to other doctors for other health issues
- ◯ I didn't have any other health problems at the time

(Select one option)

Survey Progress
58%

4.02 Have you ever accessed illicit (non-prescribed) medical cannabis?

By 'illicit medical cannabis' we mean cannabis you use for MEDICAL purposes that you have obtained without having it prescribed by a doctor.

- ◯ Yes
- ◯ No

4.02 How long ago was it that you FIRST started using illicit (non-prescribed) medical cannabis?

Please answer in YEARS

If you first started using illicit (non-prescribed) medical cannabis LESS than a year ago please enter what fraction of a year. For example, if you started illicit (non-prescribed) medical cannabis six months ago enter '0.5', if 3 months ago '0.25', if 1 month ago '0.08', if two weeks '0.04' etc.

____
(answer in years)

4.02 In the last 28 days on how many days did you use illicit (non-prescribed) medical cannabis?

If you did not use illicit (non-prescribed) medical cannabis in the last 28 days please enter '0'

____
(answer in days)

____

4.02a What is the main way you access your illicit (non-prescribed) medical cannabis?

Note: We recognise that "I grow my own" is a legal practice in the ACT.

- ◯ I grow my own
- ◯ From friends or family
- ◯ From a recreational dealer
- ◯ From a medical cannabis supplier (not prescribed by a medical practitioner)
- ◯ From a club or co-operative outlet
- ◯ From an online supplier
- ◯ From an overseas supplier
- ◯ Other

(Select one option)

4.02a What is the other main way you have accessed your illicit (non-prescribed) medical cannabis?

____

4.02a What type(s) of illicit (non-prescribed) medical cannabis do you use?

- ☐ THC only
- ☐ Mainly THC and small amounts of other cannabinoids (e.g. CBD)
- ☐ Approximately equal amounts of THC and CBD
- ☐ Mainly CBD and small amounts of other cannabinoids (e.g. THC)
- ☐ CBD only
- ☐ Unsure/Do not know
- ☐ It varies between batches

(Select all that apply)

Of the types of medical cannabis you selected in the previous question, what was the main type you used?

Note: If you only selected one option above, please select it again below.

	Select one option
THC Only	◯
Mainly THC and small amounts of other cannabinoids (e.g. CBD)	◯
Approximately equal amounts of THC and CBD	◯
Mainly CBD and small amounts of other cannabinoids (e.g. THC)	◯
CBD only	◯

4.02a What ways do you consume your illicit (non-prescribed) medical cannabis?

- ☐ Oral (tablet or capsule)
- ☐ Oral edibles (cake, cookie etc.)
- ☐ Oral liquid concentrate (oil, tincture, etc.)
- ☐ Oral mouth spray
- ☐ Nasal application (through nose)
- ☐ Smoked as a 'joint' (cigarette)
- ☐ Smoked using a 'dry' pipe (plastic, metal, glass)
- ☐ Smoked using a water pipe or 'Bong'
- ☐ Smoked by 'dabbing' or 'spotting'
- ☐ Inhaled herb using a vaporiser
- ☐ Inhaled liquid using a vaporiser
- ☐ Other

(select all that apply)

4.02a What other way have you consumed illicit (non-prescribed) medical cannabis?

____

Of the ways of consuming illicit (non-prescribed) medical cannabis you listed above, which is the main way?

Note: If you only listed one option in the last question, please select it again below

	Select one option
Oral (tablet or capsule)	◯
Oral edibles (cake, cookie etc)	◯
Oral liquid concentrate (oil, tincture etc)	◯

Oral mouth spray	◯
Nasal application	◯
Smoked as a 'joint' (cigarette)	◯
Smoked using a dry pipe (plastic, metal, glass)	◯
Smoked using a water pipe or bong	◯
Smoked by dabbing or spotting	◯
Inhaled herb using a vaporiser	◯
Inhaled liquid using a vaporiser	◯
[q04_02a_preferred_form_other]	◯

4.02a Do you feel that the quality or composition of your illicit (non-prescribed) medical cannabis is consistent over time?

- ◯ Yes
- ◯ No
- ◯ Unsure/Don't know

(Select one option)

04.02a Have you ever been unable to access your preferred source of illicit (non-prescribed) medical cannabis to treat your health concerns?

- ◯ Yes, frequently
- ◯ Yes, occasionally
- ◯ No

04.02a Are you concerned about the possibility of contaminants in your illicit (non-prescribed) medical cannabis (e.g. heavy metals, toxins, pesticides, bacteria/fungi/mould)?

- ◯ A great deal
- ◯ Quite a bit
- ◯ A little
- ◯ Not at all
- ◯ I'm not sure

(select one option)

04.02a How much does your illicit (non-prescribed) medical cannabis cost on an average week?

____

(answer in whole dollars (e.g. 20))

____

You indicated above that you have used illicit (non-prescribed) medical cannabis in the past, but that you have not accessed illicit (non-prescribed) medical cannabis in the last 28 days.

4.02b In the previous questions you indicated that you have accessed illicit (non-prescribed) medical cannabis in the past, but that you have not accessed it in the last 28 days.

Please describe the reasons you stopped using illicit (non-prescribed) medical cannabis.

- ☐ Illicit medical cannabis wasn't improving my symptoms
- ☐ Illicit medical cannabis became too expensive for me to continue
- ☐ Illicit medical cannabis became difficult to access
- ☐ I became concerned about mobile drug-testing
- ☐ I became concerned about side-effects
- ☐ I preferred using prescribed medical cannabis
- ☐ I was concerned about presence of contaminants/quality of composition
- ☐ I did not want to be involved with the illegal drug trade
- ☐ Other

(You can select more than one option.)

04.02b Please list the other reason you stopped accessing illicit (non-prescribed) medical cannabis.

____

04.02b How long ago did you stop using illicit (non-prescribed) medical cannabis?

Please enter answer in number of MONTHS

6 weeks = 1.5
3 months = 3
1 year = 12
2 years = 24
3 years = 36
10 years = 120

____
(in months (please use numbers))

____

In the previous question you indicated that you stopped accessing illicit (non-prescribed) medical cannabis less than one month ago, but in a previous question you answered that you used 0 days of illicit (non-prescribed) cannabis in the last 28 days. If you DID use illicit (non-prescribed) cannabis in the last 28 days please go back to that question and enter how many days you used. If you did NOT use illicit (non-prescribed) medical cannabis in the last 28 days please give an answer greater than '1' for the previous question?'

04.02c What was the main way you accessed your illicit (non-prescribed) medical cannabis?

Note: We recognise that "I grow my own" is a legal practice in the ACT.

- ◯ I grew my own
- ◯ From friends or family
- ◯ From a recreational dealer
- ◯ From a medical cannabis supplier (not prescribed by a medical practitioner)
- ◯ From a club or co-operative outlet
- ◯ From an online supplier
- ◯ From an overseas supplier
- ◯ Other

(Select one option)

04.02d What was the other main way you have accessed your illicit (non-prescribed) medical cannabis?

____

04.02e When you were accessing illicit (non-prescribed) medical cannabis what types did you use?

- ☐ THC only
- ☐ Mainly THC and small amounts of other cannabinoids (e.g. CBD)
- ☐ Approximately equal amounts of THC and CBD
- ☐ Mainly CBD and small amounts of other cannabinoids (e.g. THC)
- ☐ CBD only
- ☐ Unsure/Do not know
- ☐ It varies between batches

(Select all that apply)

Of the types of medical cannabis you selected in the previous question, what was the main type you used?

Note: If you only selected one option above, please select it again below.

	Select one option
THC Only	◯
Mainly THC and small amounts of other cannabinoids (e.g. CBD)	◯

Approximately equal amounts of THC and CBD	◯
Mainly CBD and small amounts of other cannabinoids (e.g. THC)	◯
CBD only	◯

04.02b When you were accessing illicit (non-prescribed) medical cannabis, what was ways did you consume it?

- ☐ Oral (tablet or capsule)
- ☐ Oral edibles (cake, cookie etc.)
- ☐ Oral liquid concentrate (oil, tincture, etc.)
- ☐ Oral mouth spray
- ☐ Nasal application (through nose)
- ☐ Smoked as a 'joint' (cigarette)
- ☐ Smoked using a 'dry' pipe (plastic, metal, glass)
- ☐ Smoked using a water pipe or 'Bong'
- ☐ Smoked by 'dabbing' or 'spotting'
- ☐ Inhaled herb using a vaporiser
- ☐ Inhaled liquid using a vaporiser
- ☐ Other

(Select all that apply)

04.02b What other way did you consume your illicit (non-prescribed) medical cannabis?

____

Of the ways of consuming illicit (non-prescribed) medical cannabis you listed above, which is the main way?

Note: If you only listed one option in the last question, please select it again below

	Select one option
Oral (tablet or capsule)	◯
Oral edibles (cake, cookie etc)	◯
Oral liquid concentrate (oil, tincture etc)	◯
Oral mouth spray	◯
Nasal application	◯
Smoked as a 'joint' (cigarette)	◯
Smoked using a dry pipe (plastic, metal, glass)	◯
Smoked using a water pipe or bong	◯
Smoked by dabbing or spotting	◯
Inhaled herb using a vaporiser	◯
Inhaled liquid using a vaporiser	◯
[q04_02b_preferred_form_other]	◯

04.02b When you were accessing illicit (non-prescribed) medical cannabis, did you feel that the quality or composition was consistent over time?

- ◯ Yes
- ◯ No
- ◯ Unsure/Don't know

(Select one option)

4.02b When you were accessing illicit (non-prescribed) medical cannabis, were you ever unable to access it to treat your health concerns?

- ◯ Yes, frequently
- ◯ Yes, occasionally
- ◯ No

4.02b When you were accessing illicit (non-prescribed) medical cannabis, were you concerned about the possibility of contaminants (e.g. heavy metals, toxins, pesticides, bacteria/fungi/mould)?

- ◯ A great deal
- ◯ Quite a bit
- ◯ A little
- ◯ Not at all
- ◯ I'm not sure

(select one option)

4.02b When you were accessing your illicit (non-prescribed) medical cannabis, how much did it cost on an average week?

If you never paid for your medical cannabis please enter '0'

____
(answer in whole dollars (e.g. 20))

____

4.03 In previous questions you indicated that you have used both legal (prescribed) and illicit (non-prescribed) medical cannabis in the last 28 days.

Please tell us what you would consider to be your MAIN source of medical cannabis (i.e. the source you use most often)?

- ◯ Legal (prescribed)
- ◯ Illicit (non-prescribed)

(Select one option.)

4.03 Please use the slider to estimate what percentage of your total medical cannabis use is prescribed?

0% 50% 100%

(Place a mark on the scale above)

____

You have said that your main source of medical cannabis is legal (prescribed) but you have said that the percentage of your total use that is prescribed is [q04_03_bothcurrent_propprescribed]%. This is contradictory. If your main source is legal (prescribed), the percentage must be 50% or more. Please think carefully and adjust your answers accordingly.

____

You have said that your main source of medical cannabis is illicit (non-prescribed) but you have said that the percentage of your total use that is prescribed is [q04_03_bothcurrent_propprescribed]%. This is contradictory. If your main source is illicit (non-prescribed), the percentage of prescribed must be 50% or less. Please think carefully and adjust your answers accordingly.

4.03 Please tell us why you use illicit (non-prescribed) MC products in addition to a legal (prescribed) MC product?

- ☐ I use different forms (prescribed or illicit) depending on supply
- ☐ I use different forms (prescribed or illicit) depending on cost
- ☐ I treat different conditions with prescribed and illicit
- ☐ Sometimes it is hard to obtain a prescription
- ☐ To improve effectiveness
- ☐ ACT only: I'm able to legally grow my own
- ☐ Other

(Select all that apply)

4.03 Please describe which other reason describes your reason for using both illicit (non-prescribed) and legal (prescribed) medical cannabis products __________________________________

In the previous question you stated that you treated different conditions with prescribed vs illicit cannabis.

For each of the conditions you stated earlier you treat with medical cannabis, please indicate whether your treat them with PRESCRIBED or ILLICIT medical cannabis.

	Prescribed	Illicit
Addiction (Cannabis)	○	○
Addiction (Other drugs: including alcohol, opioid, amphetamine)	○	○
Amyotrophic Lateral Sclerosis	○	○
Anxiety Disorder (e.g. generalised anxiety, panic disorder, OCD)	○	○
Arthritis (including rheumatoid or osteoarthritis	○	○
Attention Deficit disorder (ADHD)	○	○
Autism	○	○
Auto-Immune condition (e.g. SLE, chronic fatigue disorder)	○	○
Back pain	○	○
Blood cancers (leukaemia, lymphoma)	○	○
Brain cancers (glioblastoma, neuroblastoma, mesothelioma)	○	○
Breast cancer	○	○
Cancer-related pain	○	○
Cardiovascular condition (e.g. poor circulation, ischemic heart disease)	○	○
Circadian rhythm sleep disorder (e.g. shift work type)	○	○
Crohn's disease	○	○
Dementia (including Alzheimer's)	○	○
Depression	○	○
Diabetes Mellitus	○	○
Eating disorders (e.g. anorexia / bulimia/ obesity)	○	○
Epilepsy/ Seizure disorder	○	○
Fibromyalgia	○	○

Gastrointestinal cancer (bowel, colon, stomach)	○	○
Glaucoma	○	○
Gynaecological condition (e.g. endometriosis)	○	○
Headaches (including migraines)	○	○
HIV	○	○
Huntington's disease	○	○
Infectious disease (e.g. viral hepatitis)	○	○
Insomnia (any type)	○	○
Irritable bowel syndrome	○	○
Lung cancer	○	○
Manic Disorder (Bipolar affective disorder)	○	○
Menstrual pain	○	○
Multiple Sclerosis	○	○
Narcolepsy or other hypersomnia	○	○
Neck pain	○	○
Neuropathy (nerve pain)	○	○
[q02_01a_condition_cancer_other]	○	○
[q02_01a_condition_gastro_other]	○	○
[q02_01a_condition_mentalh_other]	○	○
[q02_01a_condition_neuro_other]	○	○
[q02_01a_condition_sleep_other]	○	○
[q02_01a_condition_pain_other]	○	○
Parasomnias (e.g. sleep walking, nightmares)	○	○
Parkinson's disease	○	○
Post-Traumatic Stress Disorder	○	○
Reproductive cancers (cervical, uterine, testicular, ovarian)	○	○
Respiratory disease (e.g. asthma, cystic fibrosis)	○	○
Schizophrenia or other psychosis	○	○
Skin cancers (melanoma)	○	○
Skin condition (e.g. eczema, psoriasis, dermatitis)	○	○
Sleep apnoea or other sleep-related breathing disorder	○	○

Sleep-related movement disorder (e.g. restless leg syndrome)	○	○
Spinal cord injury	○	○
Tourette's Syndrome	○	○
Ulcerative colitis	○	○
[q02_01a_condition_other_other]	○	○

____

4.04 You indicated in previous questions that you have used legal (prescribed) medical cannabis in the last 28 days, AND that you are a former illicit (non-prescribed) medical cannabis user (i.e. you have not used illicit medical cannabis in the last 28 days).

Please tell us why you stopped using illicit medical cannabis but have continued using prescribed medical cannabis?

- ☐ Health reasons (prescribed is healthier)
- ☐ Prescribed is more effective at treating my condition and its symptoms
- ☐ I was concerned about getting in trouble with the law for using illicitly-sourced cannabis
- ☐ Concern over possible contaminants of illicit medical cannabis
- ☐ Wanted to be sure of the content of my medical cannabis (e.g. proportion of CBD vs THC)
- ☐ I wanted to be able to receive medical advice about how best to administer my medical cannabis (e.g. advice about dose, frequency etc)
- ☐ Other

(Select all that apply)

4.04 What is the other reason you stopped accessing illicit medical cannabis but continued using prescribed medical cannabis?

____

____

4.05 You indicated in previous questions that you have used illicit (non-prescribed) medical cannabis in the the last 28 days, and that you are a former prescribed medical cannabis user (i.e. you have not used prescribed medical cannabis in the last 28 days).

Please tell us why you stopped using prescribed medical cannabis but have continued using illicit (non-prescribed) medical cannabis?

- ☐ Health reasons (illicit is healthier/more natural)
- ☐ Illicit is more effective at treating my condition and its symptoms
- ☐ Prescribed medical cannabis is too expensive
- ☐ I was having difficulty obtaining a prescription/regular prescriber for my conditions
- ☐ I was worried about stigma from health professionals
- ☐ Other

(Select all that apply)

4.05 What is the other reason you stopped accessing legal (prescribed) medical cannabis but continued using illicit (non-prescribed) medical cannabis?

____

____

4.06 You have indicated in previous answers that you have used both legal (prescribed) medical cannabis and illicit (non-prescribed) medical cannabis in the past, but that you have not used either form of medical cannabis in the last 28 days

Please tell us why you stopped using any form of medical cannabis?

- ☐ I recovered from my condition and no longer needed medical cannabis to treat it
- ☐ Cost
- ☐ I just wanted a break from cannabis use
- ☐ I was concerned about becoming dependent (i.e. addicted)
- ☐ I found the side effects were too unpleasant
- ☐ I began using other ways to treat my condition(s) (e.g. other medications, counselling, surgery, exercise, meditation)
- ☐ Cannabis stopped being effective at treating my condition
- ☐ Other

(Select all that apply)

4.06 Please tell us the other reason you stopped using both prescribed and illicit (non-prescribed) medical cannabis.

____

4.06 Do you still currently use cannabis recreationally (i.e. non-medically)?

- ◯ Yes
- ◯ No

4.07 You have indicated in previous answers that you have NEVER used illicit (non-prescribed) medical cannabis in the past.

Please tell us why you have never used any type of illicit (non-prescribed cannabis).

- ☐ I prefer using prescribed medical cannabis
- ☐ Cost
- ☐ I was concerned about its illegality
- ☐ I was concerned about contaminants
- ☐ I was concerned about the health consequences
- ☐ I didn't know how to obtain illicit cannabis
- ☐ I don't know what is in illicit cannabis (e.g. the amount of THC/CBD)
- ☐ I was worried what people would think of me using cannabis (i.e. stigma)
- ☐ Other

(Select all that apply)

4.07 Please tell us the other reason you have never used illicit (non-prescribed) cannabis.

____

____

4.08 In the previous questions you indicated that you have never used either prescribed medical cannabis or illicit (non-prescribed) medical cannabis. This means you are not eligible for the survey. Please consider your previous answers carefully and complete them again if you have made a mistake.

____

4.09 You have indicated that you have used both legal (prescribed) and illicit (non-prescribed) medical cannabis.

For each of the following please indicate whether you prefer illicit (non-prescribed) or legal (prescribed) medical cannabis

	Strongly prefer illicit	Prefer illicit	No preference	Prefer Prescribed	Strongly prefer prescribed
Consistency of dose from batch to batch	◯	◯	◯	◯	◯
Ease of access (getting supplies)	◯	◯	◯	◯	◯
Cost	◯	◯	◯	◯	◯
Effectiveness (how well it treats my health condition)	◯	◯	◯	◯	◯
Side effects	◯	◯	◯	◯	◯
Illegality (avoiding the possibility of prosecution/charge)	◯	◯	◯	◯	◯

4.10 Would you be likely to use a low-dose over-the-counter CBD product if one were available?

By 'low-dose' we mean less than 150 mg daily.

◯ Yes
◯ No
◯ Don't know

SECTION 5

CONSEQUENCES OF MEDICAL CANNABIS USE AND EXPERIENCES WITH STOPPING MEDICAL CANNABIS USE

In the next few pages we would like to hear about some of your experiences using medical cannabis, both while you are using it and when you stop (if you have stopped).

Please click 'Next Page' to start.

Survey Progress
82%

SECTION 5.01: CONSEQUENCES OF USING CANNABIS OR CANNABIS PRODUCTS

Considering your use of cannabis, please tell us whether any of the following statements have applied to you at any time in the last 12 months

	Yes	No
I often take cannabis in larger amounts or over a longer period of time than I intended to	○	○
I have a persistent desire, or make unsuccessful attempts to cut down or control my cannabis use	○	○
I spend a great deal of time in activities necessary to obtain cannabis, use cannabis, or recover from its effects	○	○
I have cravings, or a strong desire or urge to use cannabis	○	○
My cannabis use results in failure to fulfil my major role obligations at work, school, or home	○	○
I continue to use cannabis despite having persistent or recurrent social or interpersonal problems related to cannabis use (such as criminal charges, ultimatums of abandonment from spouses/partners/friends, and poor productivity)	○	○
I have given up or reduced important social, occupational, or recreational activities because of cannabis use	○	○
I recurrently used cannabis in situations in which it is physically hazardous (e.g. driving motor vehicle, operating machinery)	○	○
I continue to use cannabis even though it causes problems with emotions, mental, or physical health (e.g. persistent cough)	○	○

I needed to use cannabis more than before to get the desired effects or the same amount of cannabis has less effects than before	○	○

SECTION 5.02: EXPERIENCES WHEN STOPPING MEDICAL CANNABIS

Please tell us about your experiences stopping medical cannabis use.

Considering your use of medical cannabis in the last 12 months, please indicate whether any of the following have applied to you.

Within a week of stopping cannabis use that has been heavy or prolonged (i.e. usually daily or almost daily use over a period of at least a few months) I develop...

	Yes	No
Irritability, anger or aggression	○	○
Nervousness or anxiety	○	○
Decreased appetite or weight loss	○	○
Restlessness	○	○
Sleep difficulty (e.g., insomnia, disturbing dreams)	○	○
Depressed mood	○	○
Significant distress from at least one of the following: abdominal pain, shakiness/tremors, sweating, fever, chills or headache	○	○
I take cannabis to relieve or avoid withdrawal symptoms	○	○

5.02a In the last 12 months have you had a break of one month or more from using cannabis?

In other words, have you stopped using all types of cannabis (both medical and non-medical/recreational) for one month or more in the last 12 months?

○ Yes
○ No

5.02a Why did you stop using medical cannabis?

☐ Unable to find supply
☐ Could not afford it
☐ Did not like the side effects (including the 'high')
☐ It did not work in treating my conditions or its symptoms
☐ I recovered from my health complaint (had not more symptoms)
☐ I wanted a break from cannabis use
☐ Other
(select all that apply)

5.02a What was the other reason you stopped using medical cannabis?

____

SECTION 5.03: SIDE EFFECTS

Indicate below whether you have experienced any of the following side effects in the last 12 months when using medical cannabis, and, if so, how serious these side effects were.

Note: 'Mild and Tolerable' = symptoms that caused only mild impairment of function and do not require specific treatment (e.g. other medications or stopping medical cannabis)

'Severe and/or intolerable' = symptoms that caused impairment of function, and/or resulted in either other treatment (e.g. other medications) or resulted in stopping medical cannabis use.

	No	Mild and tolerable	Severe and/or intolerable
Allergies	◯	◯	◯
Anxiety	◯	◯	◯
Cannabis hyperemesis	◯	◯	◯
Confusion	◯	◯	◯
Constipation	◯	◯	◯
Decreased appetite	◯	◯	◯
Dehydration	◯	◯	◯
Depressed mood	◯	◯	◯
Diarrhoea	◯	◯	◯
Dizziness	◯	◯	◯
Drowsiness or sedation	◯	◯	◯
Dry mouth	◯	◯	◯
Eye irritation (red, sore or itchy eyes)	◯	◯	◯
Gastro-intestinal irritation	◯	◯	◯
Headaches	◯	◯	◯
Increased appetite	◯	◯	◯
Lack of energy or fatigue	◯	◯	◯
Memory impairment	◯	◯	◯
Nasopharyngeal complaints (e.g. sinusitis, pharyngitis)	◯	◯	◯
Nausea and/or vomiting	◯	◯	◯
Panic attack	◯	◯	◯
Paranoia/hallucinations/delusions	◯	◯	◯
Racing heart or palpitations	◯	◯	◯
Residual bad taste in mouth	◯	◯	◯
Respiratory complaints (e.g. bronchitis, cough)	◯	◯	◯

Shaking/tremor/difficulty controlling movements	◯	◯	◯
Sleep disturbance	◯	◯	◯
Sweating	◯	◯	◯
Other	◯	◯	◯

Please indicate what other type of side effect you have experienced.

Note: If you have experienced no other side effects please select 'No' as the response option for that question and this box will disappear.

____
(please describe)

Survey Progress
88%

SECTION 6

Medical Cannabis, Health Care, and Social Issues

6.01 Which of the following MOST informed your decision to use medical cannabis for your health condition?

◯ Discovered benefits on my own (i.e. was using cannabis for non-medical reasons and noticed my symptoms improved)
◯ Social media (e.g. Facebook, Reddit, Instagram)
◯ Conventional media (e.g. television, radio, newspaper, magazine)
◯ Conventional healthcare provider (e.g. GP, medical specialist, pharmacist, nurse, counsellor)
◯ Alternative healthcare practitioner (e.g. herbalist, naturopath)
◯ Friend or family member
◯ Personal research (e.g. journal articles, books, google scholar)
◯ Consumer group focused on a particular condition (e.g. epilepsy, chronic pain, mental health)
◯ Medical cannabis advocacy group (e.g. United in Compassion, Hemp embassy)
◯ Other
(select one option)

6.01a What other source most informed your decision to use medical cannabis for your condition?

____

6.02 Have you discussed your medical cannabis use with any of your healthcare providers?

◯ Yes - with most of my healthcare providers
◯ Yes - but only with a small number of my healthcare providers
◯ No - not with any of my healthcare providers
(select one option)

6.02a	Please indicate which healthcare providers you have discussed your medical cannabis use with	☐ My usual General Practitioner (GP)<br>☐ General Practitioner (GP) at a cannabis clinic<br>☐ Specialist medical practitioner (e.g. pain specialist, neurologist, oncologist, psychiatrist)<br>☐ Specialist at a cannabis clinic<br>☐ Counsellor/psychologist<br>☐ Nurse<br>☐ Pharmacist<br>☐ Physiotherapist<br>☐ Alternative therapist (e.g. acupuncturist, herbalist)<br>☐ Other<br>(select all that apply)
6.02b	Please tell us what other kind of healthcare provider you have discussed your medical cannabis use with.	__________________________________<br>(please describe)
6.02c	Which of the following best describes your healthcare provider's general attitude to your use of medical cannabis?	○ All are supportive of my medical cannabis use<br>○ Most (but not all) are supportive of my medical cannabis use<br>○ Uncertain<br>○ Most (but not all) do not support my medical cannabis use<br>○ All do not support my medical cannabis use<br>(select one option)
6.02d	Did you feel comfortable disclosing the details of your medical cannabis use to your medical practitioners?	○ Yes - to most of my medical practitioners<br>○ Yes - but only to one or a few of my medical practitioners<br>○ No
6.03	Do you feel comfortable disclosing the details of your medical cannabis use with close friends/family?	○ Yes - to most of my friends/family<br>○ Yes - but only to some close friends/family members<br>○ No<br>(select one option)
6.03a	Which of the following best describes your family and friends' general attitude to your use of medical cannabis	○ All are supportive of my medical cannabis use<br>○ Most (but not all) are supportive of my medical cannabis use<br>○ Uncertain<br>○ Most (but not all) do not support my medical cannabis use<br>○ All do not support my medical cannabis use<br>(select one option)

FINANCIAL, EMPLOYMENT, AND LEGAL IMPLICATIONS

Please indicate to what extent you agree or disagree with the following statements

	Strongly disagree	Disagree	Neither agree nor disagree	Agree	Strongly Agree
Accessing medical cannabis places a major strain on my finances	◯	◯	◯	◯	◯
Accessing medical cannabis makes me worry about being arrested or experiencing other legal problems	◯	◯	◯	◯	◯
Accessing medical cannabis makes me worry about the security of my employment	◯	◯	◯	◯	◯
Using medical cannabis makes me worry about being stigmatised by medical professionals or by my friends and family	◯	◯	◯	◯	◯

6.04 Do you ever have to do workplace drug testing?
◯ Yes
◯ No
◯ Not sure

6.04a Was your medical cannabis use detected by the test?
◯ Yes
◯ No

6.04b Were there any consequences as a result of your positive test?
◯ Yes
◯ No

6.04c What were the consequences?
____

Survey Progress
92%

ATTITUDES TO REGULATION OF MEDICAL CANNABIS

Please indicate the degree to which you agree/ disagree with each of the following statements regarding medical cannabis:

	Strongly disagree	Disagree	Neither agree or disagree	Agree	Strongly agree
A person should only be able to legally use medical cannabis if a medical professional prescribes it for them	◯	◯	◯	◯	◯
Medical cannabis should be considered as part of routine health care in Australia	◯	◯	◯	◯	◯
Medical cannabis should meet safety standards (e.g. be of known strength, composition and be free of contaminants)	◯	◯	◯	◯	◯
The cost of medical cannabis should be subsidised by the government	◯	◯	◯	◯	◯
The current regulatory framework in Australia for accessing medical cannabis works well	◯	◯	◯	◯	◯
The cost of accessing legal medical cannabis in Australia is prohibitively expensive	◯	◯	◯	◯	◯
The current model for accessing medical cannabis in Australia is 'straightforward' / 'easy to negotiate' for patients	◯	◯	◯	◯	◯
People should be able to buy low-dose CBD over the counter	◯	◯	◯	◯	◯

6.05a What should be the legal status of cannabis in Australia?

- ◯ Cannabis should be legal for ALL purposes (medical and non-medical)
- ◯ Cannabis should be legal for MEDICAL purposes ONLY
- ◯ Cannabis should be ILLEGAL for ALL reasons (including medical)
- ◯ Don't know

6.06 Since new rules were introduced around personal use of cannabis in ACT on 31 January 2020, have you started growing your own cannabis?

- ◯ Yes
- ◯ No
- ◯ I'm not aware of any new rules in ACT

Survey Progress
94%

SECTION 7

DRIVING

7.01 Have you driven a motor vehicle in the past 12 months?

- ◯ Yes
- ◯ No

7.01a Why haven't you driven a car in the last 12 months?

- ☐ I don't have a driver's licence
- ☐ I have not driven because I have been using medical cannabis
- ☐ I have not driven for other reasons (e.g. health condition, don't have a car, don't need to drive)

7.01b Please tell us why you have not driven a car in the last 12 months

____

7.02 After using medical cannabis, how long do you typically wait before driving?

- ◯ < 1 hour
- ◯ 1-3 hours
- ◯ 4-6 hours
- ◯ 7-12 hours
- ◯ 13-24 hours
- ◯ >24 hours

7.02a How long after using medical cannabis do you think you are safe to drive?

- ◯ < 1 hour
- ◯ 1-3 hours
- ◯ 4-6 hours
- ◯ 7-12 hours
- ◯ 13-24 hours
- ◯ >24 hours

7.03 In the last 12 months, have you been roadside drug tested?

- ◯ Yes
- ◯ No

7.03a What was the outcome of your roadside drug testing?

- ☐ Tested negative
- ☐ Tested positive but was not convicted (i.e., no penalties)
- ☐ Tested positive and was convicted (e.g., loss of license, fine).

(Select all that apply)

7.04 Does the presence of roadside drug testing deter you from driving after you have consumed medical cannabis?

- ◯ Yes
- ◯ No

7.04a Does the presence of roadside drug testing deter you from using medical cannabis or affect what type of medical cannabis you use?

- ◯ Yes
- ◯ No

7.05 In the past 12 months, did you ever drive while under the influence of cannabis (i.e. while you were high)?

- ◯ Yes
- ◯ No

7.05a In the past 12 months, how often did you drive while under the influence of cannabis (i.e. while you were high)?

- ◯ Very rarely
- ◯ Rarely
- ◯ Sometimes
- ◯ Often
- ◯ Very often

(Select one option)

7.05b What were some of the reasons you drove while high?

- ☐ I didn't think I was impaired
- ☐ I thought I was impaired but could still drive safely
- ☐ I didn't have an alternative transport option (e.g. public transport, uber, taxi)
- ☐ I had another transport option but it was a hassle (e.g. had to wait too long, too expensive)
- ☐ another reason

7.05bi What was the other reason you drove while high?

____

Of the reasons for driving while high you listed above, what was the main reason?

If you only listed one reason please check the box again below

	Select one option
I didn't think I was impaired	○
I thought I was impaired but could still drive safeily	○
I didn't have an alternative transport option (e.g. public transport, uber, taxi)	○
I had another transport option but it was a hassle (e.g. wait time too long, too expensive)	○
Another reason	○

Thinking about your driving and how you feel after consuming medical cannabis, please indicate to what extent you agree or disagree with the following statements:

	Strongly disagree	Disagree	Neither agree nor disagree	Agree	Strongly agree
I am slower to react to sudden situations	○	○	○	○	○
I find it harder to remain focused	○	○	○	○	○
I tend to drive more carefully	○	○	○	○	○
I find it harder to stick to the speed limit	○	○	○	○	○
I tend to leave a larger gap between myself and the car ahead	○	○	○	○	○
I find myself taking more risks	○	○	○	○	○
I find it harder to drive in a straight line	○	○	○	○	○
I feel more in control of the vehicle	○	○	○	○	○
I can accurately assess my driving ability	○	○	○	○	○

7.07 Do you think medical cannabis impairs your driving ability?

- ○ Yes
- ○ No

(select one option)

7.08 Do you think non-medical ('recreational') cannabis impairs your driving ability?

- ○ Yes
- ○ No

Survey Progress
98%

SECTION 8

GENERAL HEALTH AND QUALITY OF LIFE

The following questions ask you about your general health and quality of life.

8.01 In general would you say your health is...

- ○ Excellent
- ○ Very good
- ○ Good
- ○ Fair
- ○ Poor

8.02 In general would you say your quality of life is...

- ○ Excellent
- ○ Very good
- ○ Good
- ○ Fair
- ○ Poor

8.03 In general, how would you rate your physical health?

- ◯ Excellent
- ◯ Very good
- ◯ Good
- ◯ Fair
- ◯ Poor

8.04 In general, how would you rate your mental health, including your mood and your ability to think?

- ◯ Excellent
- ◯ Very good
- ◯ Good
- ◯ Fair
- ◯ Poor

8.05 In general, how would you rate your satisfaction with your social activities and relationships?

- ◯ Excellent
- ◯ Very good
- ◯ Good
- ◯ Fair
- ◯ Poor

8.06 To what extent are you able to carry out your everyday physical activities such as walking, climbing stairs, carrying groceries, or moving a chair?

- ◯ Completely
- ◯ Mostly
- ◯ Moderately
- ◯ A little
- ◯ Not at all

8.07 In the last 7 days how would you rate your pain on average?

0 No pain — 10 Worst pain imaginable

(Place a mark on the scale above)

8.08 In the last 7 days how would you rate your fatigue on average?

- ◯ None
- ◯ Mild
- ◯ Moderate
- ◯ Severe
- ◯ Very severe

8.09 In the last 7 days, in general, please rate how well you have carried out your usual social activities and roles.

(This includes activities at home, at work, and in your community, and responsibilities as a parent, child, spouse, employee, friend etc.)

- ◯ Excellent
- ◯ Very good
- ◯ Good
- ◯ Fair
- ◯ Poor

8.10 In the last 7 days, how often have you been bothered by emotional problems such as feeling anxious, depressed, or irritable?

- ◯ Never
- ◯ Rarely
- ◯ Sometimes
- ◯ Often
- ◯ Always

Survey Progress
99%

END OF SURVEY

Thank you so much for taking the time to complete this survey.

Is there anything we have not asked that you would like to add in regards to your experience of medical cannabis? (Please do not disclose identifying information such as your name)

When you are finished please click the 'submit' button.

____

Since 2020 ACT residents are allowed by law to possess and grow small amounts of cannabis.

At the start of the survey you indicated that you live in the ACT.

If you are interested in taking part in a 10-minute survey about cannabis use in the ACT, please go to The CAN-ACT survey

(But don't forget to click the 'Submit' button on this survey :)
